# Mucosal immunoglobulins at respiratory surfaces mark an ancient association that predates the emergence of tetrapods

**DOI:** 10.1038/ncomms10728

**Published:** 2016-02-12

**Authors:** Zhen Xu, Fumio Takizawa, David Parra, Daniela Gómez, Louise von Gersdorff Jørgensen, Scott E. LaPatra, J. Oriol Sunyer

**Affiliations:** 1 Department of Pathobiology, School of Veterinary Medicine, University of Pennsylvania, 413 Rosenthal building, 3800 Spruce Street, Philadelphia, Pennsylvania 19104, USA; 2 Department of Aquatic Animal Medicine, College of Fisheries, Huazhong Agricultural University, Wuhan, Hubei 430070, China; 3 Department of Cell Biology, Physiology and Immunology, Universitat Autònoma de Barcelona, Barcelona 08193, Spain; 4 Laboratory of Aquatic Pathobiology, Department of Veterinary Disease Biology, Faculty of Health and Medical Sciences, University of Copenhagen, Frederiskberg DK-1870, Denmark; 5 Research Division, Clear Springs Foods Inc., P O Box 712, Buhl, Idaho 83316, USA

## Abstract

Gas-exchange structures are critical for acquiring oxygen, but they also represent portals for pathogen entry. Local mucosal immunoglobulin responses against pathogens in specialized respiratory organs have only been described in tetrapods. Since fish gills are considered a mucosal surface, we hypothesized that a dedicated mucosal immunoglobulin response would be generated within its mucosa on microbial exposure. Supporting this hypothesis, here we demonstrate that following pathogen exposure, IgT^+^ B cells proliferate and generate pathogen-specific IgT within the gills of fish, thus providing the first example of locally induced immunoglobulin in the mucosa of a cold-blooded species. Moreover, we demonstrate that gill microbiota is predominantly coated with IgT, thus providing previously unappreciated evidence that the microbiota present at a respiratory surface of a vertebrate is recognized by a mucosal immunoglobulin. Our findings indicate that respiratory surfaces and mucosal immunoglobulins are part of an ancient association that predates the emergence of tetrapods.

## Abstract (web summary)

In teleost fish the gills perform—in addition to respiration—functions such as immune defence. Here the authors show that IgT, a teleost specific Ig previously shown to be involved in gut and skin mucosal immunity, is locally induced in the gill, where it plays a key role in immunity in rainbow trout.

## Graphical abstract

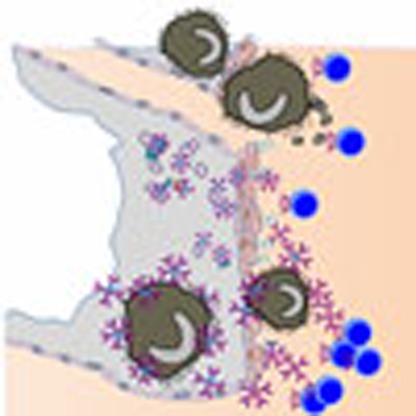

Individual vertebrate species have faced unique evolutionary pressures to continuously acquire oxygen from the environment, which has led to the development of diverse gas-exchange structures (that is, respiratory organs)^1^. In most vertebrates, respiration occurs through specialized gas-exchange surfaces (that is, gill, lungs), although a small percentage of gas exchange may also occur through the skin (that is, cutaneous respiration)^2^. Design of specialized respiratory organs has evolved in response to several factors, most critically the environment where the organism lives, but also the size and phylogenetic position of the organism^1^. In general, vertebrates living in aqueous media have evolved evaginated gas-exchanges structures (that is, gills) while those acquiring oxygen from air have developed invaginated structures (that is, lungs) to prevent desiccation. An effective gas-exchange organ needs to maximize the efficiency with which oxygen from the external media (water/air) comes in contact with blood. In the case of teleost fish this is accomplished by four pairs of vascularized gill arches composed of hundreds of gill filaments, which increase their contact surface by folding into the secondary lamella. In addition to respiration, gills perform other functions including osmoregulation, pH balance, ammonia excretion, hormone regulation, detoxification and immune defense^1^. Gills are in direct contact with the water and therefore are continuously exposed to environmental toxins and pathogens. Thus, there is an evident need for the fish to defend such a large and delicate surface from pathogenic attack. While little is known about how gill immunity operates, it has been reported that gill tissue contains a significant number of innate and adaptive immune cells and that several innate and adaptive immune molecules and pathways operate in the gill^3^,^4^,^5^,^6^.

Teleost fish contain three immunoglobulin classes IgM, IgD and IgT/Z^7^. While IgM represents the most abundant class, strong IgM immune responses to infection or vaccination are mainly detected in plasma, whereas IgM titres in mucosal tissues such as gut, skin or gills remain low^7^. Moreover, IgM has been shown to coat a significant portion of the trout skin, gut and nasal microbiota^8^,^9^,^10^. Secreted IgD has been identified in catfish and trout plasma^11^,^12^, although its function in teleosts still remains undetermined. As opposed to trout secretory IgD, the secreted form of catfish IgD lacks a V region and it has been suggested that this immunoglobulin may function as an innate pattern recognition molecule^11^. In addition to IgM and IgD, teleosts contain a third immunoglobulin class, IgT (also called IgZ), first identified at the genome level in 2005 (refs 13, 14). Except catfish and medaka, all other teleost species studied express this immunoglobulin^15^. We have previously reported that teleost IgT is an immunoglobulin specialized for gut and skin mucosal immunity^8^,^9^. More specifically, we showed that IgT is the main immunoglobulin induced in the gut and skin mucosa on pathogenic challenge, and we also demonstrated that IgT plays a prevalent role in the coating of the microbiota found in these surfaces. Moreover, we discovered a novel B-cell lineage uniquely expressing surface IgT which represents the predominant B-cell subset in the trout gut-, skin- and nasal-associated lymphoid tissues (GALT, SALT and NALT)^8^,^9^,^10^. Overall, fish IgT represents the most ancient mucosal immunoglobulin found in a vertebrate species. In fact, the newly attributed role of IgT in mucosal immunity has challenged the previous paradigm that specialization of immunoglobulin isotypes into mucosal and systemic responses arose during tetrapod evolution^8^.

Three B-cell subsets have been identified in catfish^11^: IgM^+^IgD^−^, IgM^+^IgD^+^, IgM^−^IgD^+^, while generally two B-cell subsets can be identified in all other species that contain IgT/IgZ, including the IgM^+^IgD^+^IgT^−^ and the IgM^−^IgD^−^IgT^+^ (ref. 12). Moreover, one study has recently described a new population of trout IgD^+^ B cells devoid of surface IgM expression (IgD^+^IgM^−^ B cells), which was shown to represent a major B-cell subset in gill^16^. Since the same antibodies used in the aforementioned study had failed to detect these IgD^+^IgM^−^ B cells in a previous study^12^, it was suggested that this B-cell subset might only be present in an European trout line^16^.

Gills are considered a mucosal surface, and through convergent evolution they have evolved structures and components similar to those of the mammalian respiratory mucosa^1^. In this regard, the epithelia of gill primary and secondary lamella is covered by one to four layers of cuboidal or squamous cell epithelia^17^, similar to what it is found in the upper respiratory tract of mammals. Another similarity between these surfaces is the existence of local mucus-secreting cells (that is, goblet cells), which are characteristic of type I mucosal epithelia^18^, and represent the first line of defense and cell protection. In the lungs, which represent a type I mammalian mucosal surface, secretory IgA (sIgA) is produced locally and is a key immunoglobulin involved in mucosal respiratory immunological responses^19^,^20^,^21^. A number of reports have described the induction of gill pathogen-specific IgM titres on pathogenic challenge or vaccination^22^,^23^,^24^,^25^, while antigen-specific responses mediated by other teleost immunoglobulins are thus far unknown. Since IgT in fish plays a key role in mucosal immunity in the gut and skin, it is conceivable that IgT plays a pivotal role in gill immunity. However, it has recently been suggested that trout IgD may play an important function in gill immune responses^12^,^16^,^24^. Thus, the role of the different teleost immunoglobulins in the gill remains to be clearly delineated.

Thus far, local mucosal immunoglobulin responses in specialized respiratory surfaces have only been described in tetrapods. This study was therefore undertaken to examine whether a primordial association between gas-exchange structures and specialized mucosal immunoglobulin responses exists in species preceding the tetrapods. To this end, here we examined whether dedicated mucosal B-cell and immunoglobulin responses were present in the gills of the rainbow trout, a model species in the field of evolutionary and comparative immunology^26^,^27^. The findings here support our hypothesis that IgT is the main gill immunoglobulin involved in pathogen-specific immune responses while we found no evidence of a gill IgD^+^IgM^−^ B-cell subset or pathogen-specific IgD responses. For the first time in a vertebrate species we show that the microbiota associated with a respiratory organ is recognized by a mucosal immunoglobulin, as evidenced here by the prevalent coating of gill microbiota with IgT. In addition, we demonstrate the local generation of IgT and proliferation of IgT^+^ B cells on pathogenic challenge, thus providing the first demonstration that dedicated mucosal immunoglobulins are locally induced at the mucosal surface of a non-mammalian species.

## Results

### Immunoglobulins and B cells in the gill of trout

**Figure 1:**
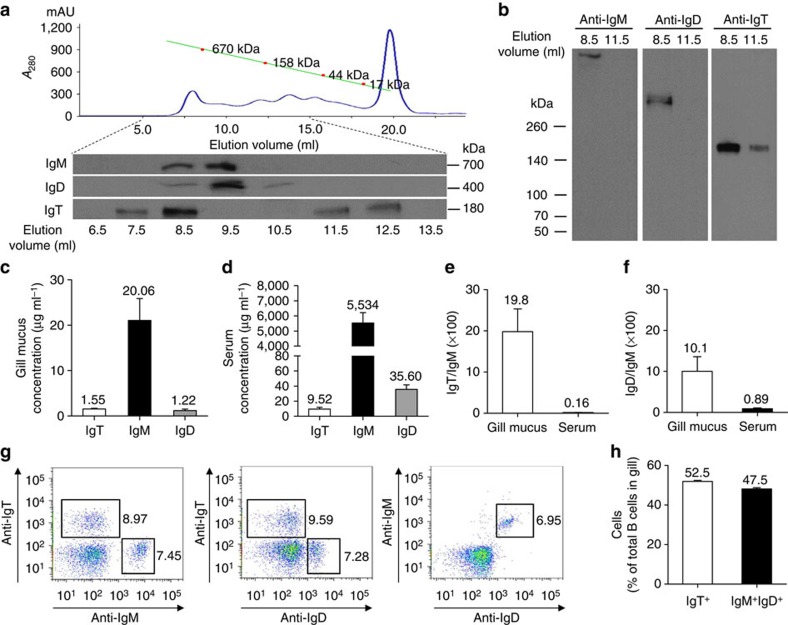
Protein characterization of gill mucus immunoglobulins and identification of B cells in the gill of trout. (**a**) Fractionation of gill mucus (∼0.5 ml) by gel filtration (upper) followed by immunoblot analysis of the fractions with anti-trout IgM, anti-trout IgD and anti-trout IgT-specific mAbs (lower). *A*_280_, absorbance at 280 nm. (**b**) SDS–PAGE of gel-filtration fractions (4–15%) corresponding to elution volumes of 8.5 and 11.5 ml under non-reducing conditions followed by immunoblot analysis with mAbs to trout IgM, IgD or IgT. (**c**,**d**) Immunoblot and densitometric analysis of the concentration of IgT, IgM and IgD in gill mucus (**c**) and serum (**d**) (*n*=15 fish). (**e**,**f**) Ratio of IgT to IgM concentration (**e**) and IgD to IgM concentration (**f**) in gill mucus and serum, calculated from the values shown in **c**,**d**. (**g**) Dot plots of leucocytes from gills. Numbers adjacent to outlined areas indicate percentage IgT^+^ B cells, IgM^+^ B cells or IgD^+^ B cells in the lymphocyte gate. (**h**) Frequency of IgT^+^ and IgM^+^IgD^+^ B cells among total B cells. Results in **c**–**e** and **h** are expressed as mean and s.e.m. obtained from 15 individual fishes.

Using an anti-IgT antibody, we detected by western blotting a protein in gill mucus consistent with the reported molecular mass of IgT^8^,^9^,^14^. To identify this immunoreactive band, the protein was purified using an anti-IgT affinity column and liquid chromatography-tandem mass spectrometry (LC/MS–MS) confirmed that the purified protein corresponded to IgT (Supplementary Fig. 1). Gel-filtration analysis showed that a majority of gill mucus IgT are present in polymeric form, as it eluted at a fraction similar to that of trout mucosal IgM, a tetrameric immunoglobulin (Fig. 1a). Interestingly, a portion of gill mucus IgT consistently eluted in monomeric form. As opposed to polymeric IgT, IgD eluted as a monomer at the molecular weight range previously reported for serum IgD^12^. Gill mucus polymeric IgT migrated as a monomer by SDS–PAGE under non-reducing conditions, indicating that polymeric IgT is associated by non-covalent interactions (Fig. 1b, right panel). As expected, mucosal IgD and IgM migrated as a monomer and a polymer, respectively, under the same SDS–PAGE conditions (Fig. 1b, left and middle panels). Next, we found that although the protein concentration of IgT was ∼13- and ∼581-fold lower than that of IgM in gill mucus and serum, respectively, (Fig. 1c,d), the ratio IgT/IgM was ∼124-fold higher in the gill mucus when compared with that of serum (Fig. 1e). A similar situation occurs with IgD, which displayed the same concentration as IgT in mucus, while its serum concentration was ∼4 times more than that of IgT and ∼155 times less than that of IgM (Fig. 1c,d). The ratio IgD/IgM was ∼10-fold greater in gill mucus than in serum (Fig. 1f). The abundance of B cells in the gills was analysed by flow cytometry. Two main B-cell subsets were detected in the gills (Fig. 1g), one expressing only surface IgT (IgT^+^ B cells), and the second one expressing both surface IgD and IgM (IgD^+^IgM^+^ B cells). From these two populations, IgT^+^ B cells were more abundant in gills (52.5% of all B cells) (Fig. 1h), suggesting a role of this subset in gill immunity. For comparison, the same B-cell subsets were found in the trout NALT and spleen (Supplementary Fig. 2), where IgT^+^ and IgM^+^ B cells are the predominant NALT and spleen B-cell subset, respectively, as previously reported^10^.

### IgT constitutes the main Ig isotype coating the gill microbiota

**Figure 2:**
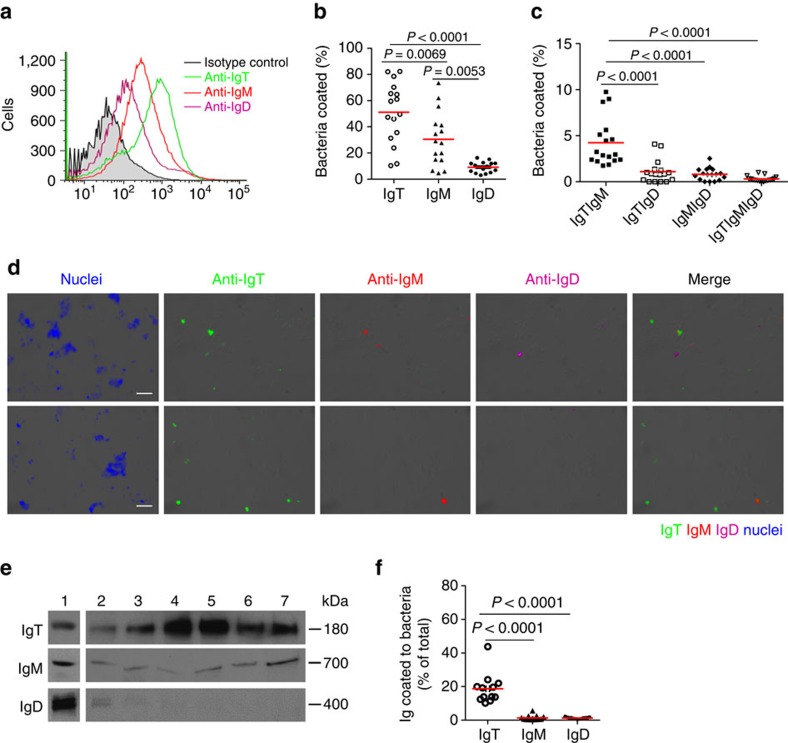
Trout gill bacteria are predominantly coated with IgT. (**a**) Representative flow cytometry histograms showing the staining of gill bacteria with IgT, IgM and IgD. Bacteria were stained with anti-trout IgT (green line), anti-trout IgM (red line) or anti-trout IgD (magenta line) mAbs or isotype controls (shaded histograms). (**b**,**c**) Percentage of gill bacteria coated with IgT, IgM or IgD (**b**) or coated with IgT and IgM, IgT and IgD, IgM and IgD, IgT and IgM and IgD (*n*=16) (**c**). The median percentage is shown by a red line. Statistical differences were evaluated by the nonparametric Mann–Whitney test. (**d**) Differential interference contrast (DIC) images of gill bacteria stained with a DAPI-Hoeschst solution (blue), anti-IgT (green), anti-IgM (red) or anti-IgD (magenta), and merging IgT, IgM and IgD stainings (Merge). (Isotype-matched control antibody staining is shown in Supplementary Fig. 3 online). (Scale bars, 5 μm). (**e**) Immunoblot analysis of IgT, IgM and IgD on gill bacteria. Lane 1, 0.1 μg of purified IgT, IgM or IgD; lanes 2–7, gill bacteria (*n*=6 fish). (**f**) Percentage of total gill mucus IgT, IgM or IgD coating gill bacteria (*n*=13). The median is shown by a red line. Statistical differences were evaluated by one-way ANOVA with Bonferroni correction. Data are representative of at least three independent experiments. ANOVA, analysis of variance.

Similar to the situation in mammals for sIgA^28^,^29^, we have previously reported that a large fraction of the gut and skin bacterial microbiota are coated with IgT, and to a lesser extent with IgM^8^,^9^. Here we adapted the methodology, which we had used to assess immunoglobulin coating of trout gut and skin bacteria^8^,^9^, to the gill mucosa, and we extended it to the analysis of IgD coating. By flow cytometry, we detected that a large fraction of gill microbiota were coated with IgT (∼51%), whereas a smaller proportion stained for IgM (∼31%) or IgD (∼10%) (Fig. 2a,b). Interestingly, we found that ∼4.23% of gill microbiota were double-coated with IgT and IgM and to a much lesser extent were double-coated with IgT and IgD (∼1.09%), IgM and IgD (∼0.91%) or triple-coated with the three immunoglobulins (∼0.19%) (Fig. 2c). Similar results were obtained by immunofluorescence microscopy (Fig. 2d; isotype-matched control antibodies, Supplementary Fig. 3). Western blot analysis further demonstrated the presence of the three immunoglobulins on gill bacteria (Fig. 2e). Notably, we found that more than 19% of total IgT present in gill mucus was used for bacterial coating, while only ∼1.5% of gill mucosal IgM and IgD was being utilized for that purpose (Fig. 2f).

### Pathogen-specific immunoglobulin responses in the gill

**Figure 3:**
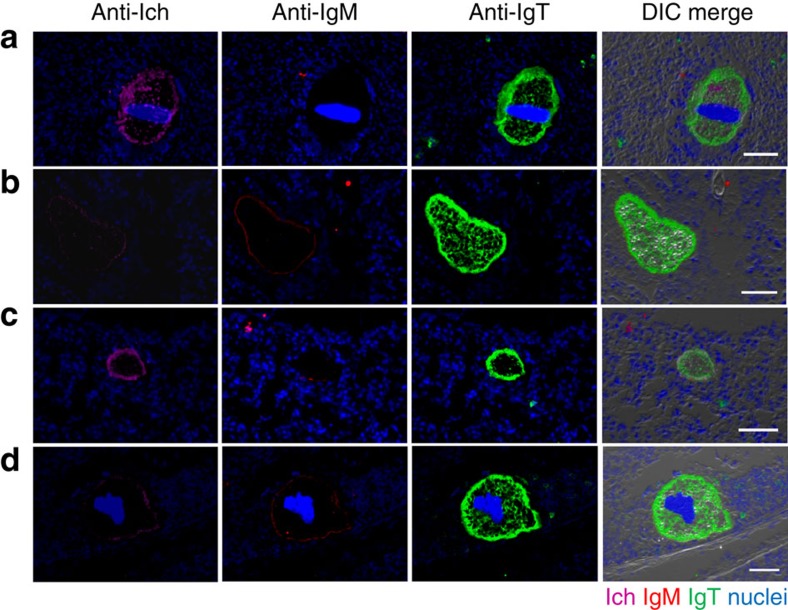
Parasites are mainly coated by IgT in the gill of infected trout. Four different microscope images (**a**–**d**) of slides showing immunofluorescence staining of Ich parasites in gill cryosections from trout infected with Ich after 25 days (*n*=6). From left to right: Ich (magenta), IgM (red) and IgT (green) with nuclei stained with DAPI (blue); DIC images showing merged staining (isotype-matched control antibody staining, Supplementary Fig. 4a online). Scale bars, 20 μm.

**Figure 4:**
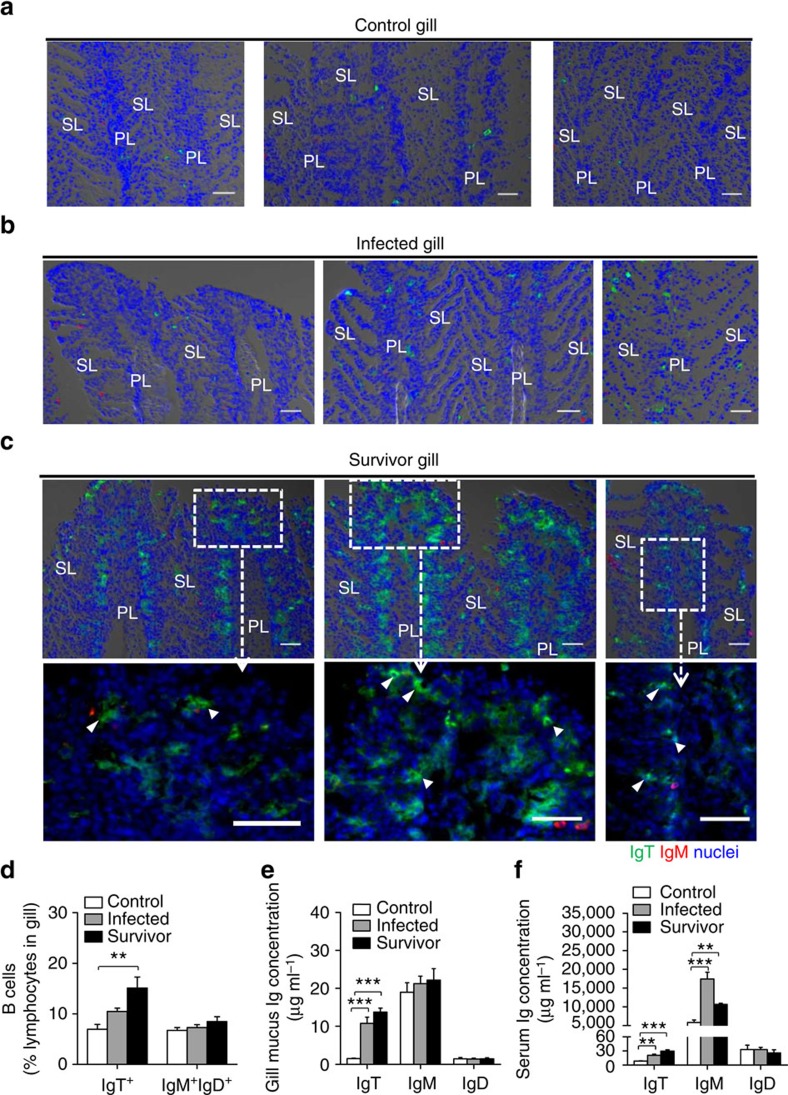
Increases in IgT^+^ B cells in the gill of trout infected with Ich. (**a**–**c**) DIC images of immunofluorescence staining on trout gill cryosections from uninfected fish (**a**), 25 days infected fish (**b**) and survivor fish (**c**, upper), stained for IgT (green) and IgM (red); nuclei are stained with DAPI (blue) (isotype-matched control antibody staining, Supplementary Fig. 4b online). Enlarged images of the areas outlined in **c** are showing some IgT^+^ B cells possibly secreting IgT (white arrowhead, **c**, lower). Primary lamellae (PL) and secondary lamellae (SL) are shown. Scale bars, 20 μm. Data are representative of at least three different independent experiments (*n*=8–9 per group). (**d**) Percentage of gill IgT^+^ and IgM^+^IgD^+^ B cells in the lymphocyte gate of uninfected control fish, infected fish and survivor fish measured by flow cytometric analysis. (**e**,**f**) Concentration of IgT, IgM and IgD in gill mucus (**e**) and serum (**f**) of control, infected and survivor fish (*n*=12 per group). **P<0.01 and ***P<0.001 (one-way ANOVA with Bonferroni correction). Data in **d**–**f** are representative of at least three independent experiments (mean and s.e.m.). ANOVA, analysis of variance.

The prevalence of IgT^+^ B cells in the gill of trout and the predominant coating of gill bacteria by IgT led us to hypothesize an important role of IgT in gill mucosal immunity. However, a role for IgD and a putative subset of IgD^+^/IgM^−^ B cells have been suggested in the gills of teleosts^12^,^16^,^24^. To evaluate which immunoglobulin played a dominant role in gill immunity, we assessed immunoglobulins and B-cell responses in fish exposed to a parasite, *Ichthyophthirius multifiliis* (Ich), that has a tropism for the skin and gills of salmonids^30^. After infection, we could detect the presence of Ich trophonts in the gills of infected fish by immunofluorescence microscopy (Fig. 3) using an anti-Ich antibody^9^. Strikingly, 25 days post infection most gill parasites were overwhelmingly recognized by IgT, while only some parasites were slightly coated with IgM (Fig. 3a–d; isotype-matched control antibodies, Supplementary Fig. 4a). However, we could never detect parasites coated by IgD (Supplementary Fig. 5). Immunofluorescent micrographs of gill samples showed very few IgT^+^ and IgM^+^ B cells in the gill filaments of control fish (Fig. 4a). However, 25 days post infection, a moderate (∼3.1-fold) but significant increase of IgT^+^ B cells was observed in the gills of infected fish (Fig. 4b). Notably, survivor fish (3 months post infection) contained large accumulations of IgT^+^ B cells in the gill filaments when compared with those of control fish (Fig. 4c; isotype-matched control antibodies, Supplementary Fig. 4b). It is worth mentioning that IgT^+^ B cells were noticeably accumulated in the primary lamella, while a few were present in the secondary lamella of survivor fish (Fig. 4c). In contrast, the number of IgM^+^ B cells did not change in the infected and survivor fish when compared with the controls (Fig. 4a–c; isotype-matched control antibodies, Supplementary Fig. 4b). Interestingly, we observed that some IgT^+^ B cells appeared to be secreting IgT (Fig. 4c, white arrows) and we noted an increase in cell size within the IgT^+^ population (Supplementary Fig. 6), which suggested the presence of IgT-producing plasmablasts- or plasma-like cells. Results from flow cytometry of B-cell populations in gills from control, infected and survivor fish mirrored those obtained by immunofluorescence microscopy. Thus, it was observed that ∼6.9±2.4% of cells from the lymphocyte gate were IgT^+^ B cells in the gills of control fish whereas a significant increase of IgT^+^ B cells was found in infected (∼10.5±2.5%) and survivor fish (∼15.2±3.7%) (Fig. 4d). In contrast, the percentage of IgM^+^ B cells did not change in any fish group (Fig. 4d). Along with this increment of IgT^+^ B cells observed in gills of infected and survivor fish, the IgT protein concentration in the gill mucus of these fish groups increased by ∼7- and ∼10-fold, respectively, when compared with control fish, while the levels of IgM and IgD remained unchanged (Fig. 4e). In serum, only approximately two and threefold increase of IgT concentration was detected in infected and survivor fish, respectively. The concentration of serum IgM increased approximately three and twofold in infected and survivor fish, respectively, when compared with control fish (Fig. 4f). In contrast, the concentration of IgD did not change significantly either in the gill mucus or serum of the same fish groups (Fig. 4e,f).

**Figure 5:**
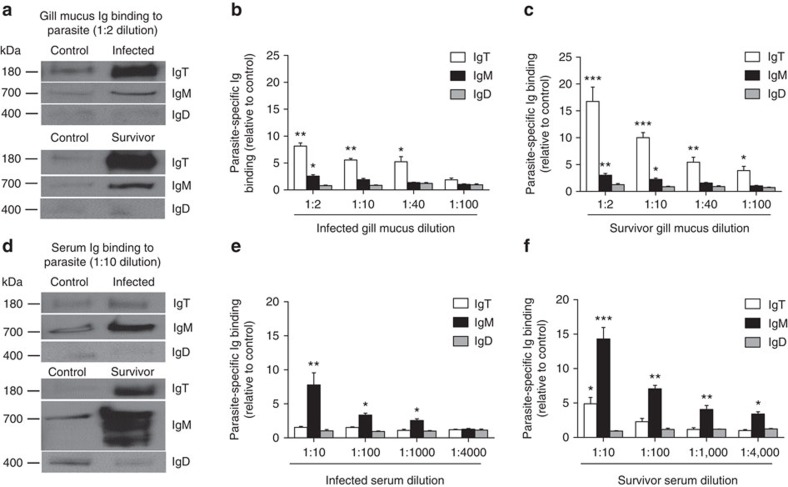
Immunoglobulin responses in the gill mucus and serum from infected and survivor fish. (**a**) Western blot analysis of IgT-, IgM- and IgD-specific binding to Ich in gill mucus (dilution 1:2) from infected and survivor fish. (**b**,**c**) IgT-, IgM- and IgD-specific binding to Ich in dilutions of gill mucus from infected (**b**) and survivor (**c**) fish, evaluated by densitometric analysis of immunoblots and presented as relative values to those of control fish (*n*=9 per group). (**d**) Western blot analysis of IgT-, IgM- and IgD-specific binding to Ich in serum (dilution 1:10) from infected and survivor fish. (**e**,**f**) IgT-, IgM- and IgD-specific binding to Ich in dilutions of serum from infected (**e**) and survivor (**f**) fish, evaluated by densitometric analysis of immunoblots and presented as relative values to those of control fish (*n*=9 per group). *P<0.05, **P<0.01 and ***P<0.001 (unpaired Student's *t*-test). Data are representative of at least three independent experiments (mean and s.e.m.).

The large increases in IgT^+^ B cells and IgT protein levels in the gills of infected and survivor fish suggested the generation of IgT-specific immune responses. Supporting this prediction, we detected significant increases in parasite-specific IgT binding in up to 1/40 and 1/100 diluted gill mucus of infected and survivor fish, respectively, in which we found ∼5.2- and ∼3.9-fold binding increases, respectively, when compared with that of control gill mucus (Fig. 5a–c). In serum, however, parasite-specific IgT binding was detected only at the 1/10 serum dilution of the survivor group. In contrast, parasite-specific IgM binding were observed in up to 1/1,000 and 1/4,000 serum dilutions from infected and survivor fish, respectively (Fig. 5d–f). We could not detect any parasite-specific IgD binding either in the gill mucus or serum from infected and survivor fish (Fig. 5a–f).

To further support that IgT is the predominant immunoglobulin induced in the gill mucosa on pathogenic challenge, we also infected fish with *Flavobacterium columnare*, a bacterial pathogen that infects the gills of salmonids. We detected approximately fivefold increase in bacteria-specific IgT binding in 1/10 diluted gill mucus of infected and survivor fish, when compared with the same dilution of control gill mucus (Supplementary Fig. 7a–c). In serum, bacteria-specific IgT binding was detected at the 1/10 serum dilution of the infected and survivor group. However, bacteria-specific IgM binding was observed in up to 1/100 serum dilution from infected and survivor fish (Supplementary Fig. 7d–f). Similar to what we found in Ich, we could not detect any bacteria-specific IgD binding in the gill mucus and serum from infected and survivor fish (Supplementary Fig. 7a–f).

### Local proliferative and immunoglobulin responses

**Figure 6:**
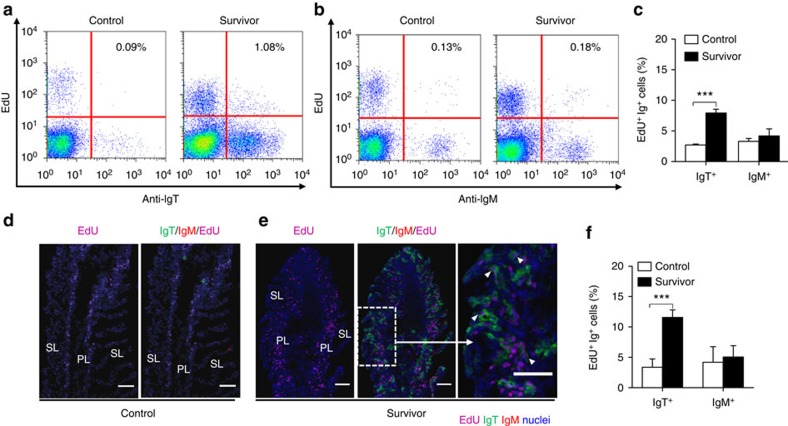
Proliferative responses of IgT^+^ and IgM^+^ B cells in the gill of fish that survived Ich infection. (**a**,**b**) Representative flow cytometry dot plot showing proliferation of IgT^+^ B cells (**a**) and IgM^+^ B cells (**b**) in gill leucocytes of control and survivor fish. The percentage of lymphocytes representing proliferative B cells (EdU^+^) is shown in each dot plot. (**c**) Percentage of EdU^+^ cells from the total gill IgT^+^ or IgM^+^ B-cell populations in control or survivor fish (*n*=12). (**d**,**e**) Immnofluorescence analysis of EdU incorporation by IgT^+^ or IgM^+^ B cells in gill of control (**d**) and survivor fish (**e**). Gill cryosections were stained for EdU (magenta), trout IgT (green), trout IgM (red) and nuclei (blue) detection (*n*=9). Primary lamellae (PL) and Secondary lamellae (SL) are shown. White arrowheads point to cells double stained for EdU and IgT. Scale bars, 20 μm. Data are representative of at least three different independent experiments. (**f**) Percentage of EdU^+^ cells from the total gill IgT^+^ or IgM^+^ B-cell populations in control or survivor fish counted from **d**,**e**. Data in **c** and **f** are representative of at least three independent experiments (mean and s.e.m.). Statistical analysis was performed by unpaired Student's *t*-test. ***P<0.001.

**Figure 7:**
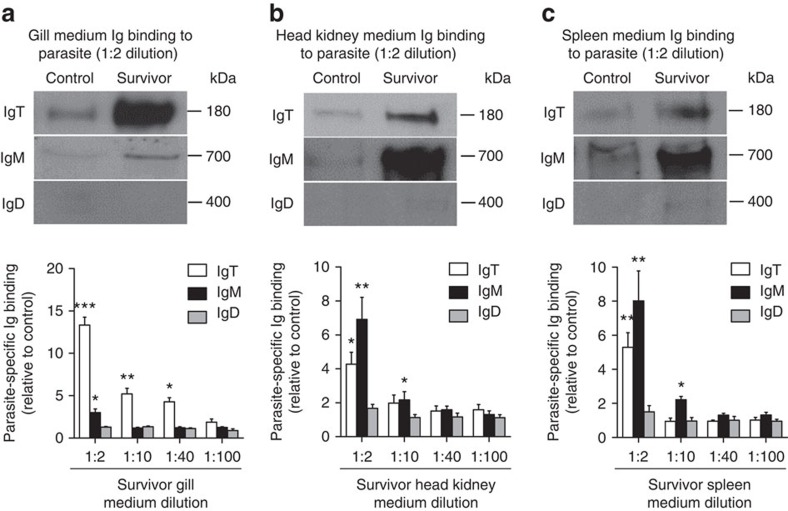
Local IgT-, IgM- and IgD-specific responses in gill explants of survivor fish. Gill, head kidney and spleen explants (∼50 mg each) from control and survivor fish were cultured for 7 days. (**a**–**c**) Western blot analysis of IgT-, IgM- and IgD-specific binding to Ich in the culture medium of gill (**a**, upper), head kidney (**b**, upper) and spleen (**c**, upper) (dilution 1:2) from control and survivor fish. (**a**–**c**) IgT-, IgM- and IgD-specific binding to Ich in dilutions of culture medium from gill (**a**, lower), head kidney (**b**, lower) and spleen (**c**, lower) from control and survivor fish, measured by densitometric analysis of immunoblots and presented as relative values to those of control fish (*n*=9–12 per group). *P<0.05, **P<0.01 and ***P<0.001 (unpaired Student's *t*-test). Data are representative of at least three independent experiments (mean and s.e.m.).

The substantial increase of IgT^+^ B cells observed in the gill of survivor fish could be due to an infiltration of these cells from systemic lymphoid organs or to a process of local IgT^+^ B proliferation. To clarify this point, we assessed the *in vivo* proliferative responses of B cells on treatment of control and survivor fish with fluorescent EdU (5-ethynyl-2′-deoxyuridine, Alexa Fluor 488), a thymidine analogue that incorporates into DNA during cell division^31^. By flow cytometry, we detected an overall increase in proliferation (∼2.6-fold) within the whole gill leucocyte population of survivor fish when compared with that of control animals (Supplementary Fig. 8). Interestingly the largest increase in proliferation occurred within the B-cell population (∼8.8-fold), followed by the large cells (∼2.6-fold) and the non-B-lymphocyte (∼2.4-fold) subsets. Within the B-lymphocyte population, we detected a significant increase in the percentage of proliferating IgT^+^ B cells (EdU^+^IgT^+^ B cells) in the gills of survivor fish (∼8.1±1.8% of all IgT^+^ B cells) when compared with that of control animals (∼2.5±0.77% of all IgT^+^ B cells) (Fig. 6a,c). However, no differences were detected in the percentage of EdU^+^IgM^+^ B cells between control and survivor fish (Fig. 6b,c). Immunofluorescent microscopic analysis of gills from EdU-injected fish yielded results very similar to those obtained by flow cytometry. Thus, we found an approximately fourfold increase in the number of EdU^+^IgT^+^ B cells in survivor fish when compared with that of control fish, while no differences in proliferating IgM^+^ B cells were detected between these two groups (Fig. 6d–f). Systemic lymphoid tissues of survivor fish, however, did not show increased IgT^+^ B-cell proliferative responses when compared with control animals, while a rise of EdU^+^IgM^+^ B cells was detected in head kidney of survivors (Supplementary Fig. 9). These results suggested that the parasite-specific IgT responses shown in Fig. 5 were locally generated in the gills rather than being produced and transported from systemic lymphoid organs. To test this hypothesis, we analysed parasite-specific immunoglobulin titres from medium derived of cultured gill, head kidney and spleen explants obtained from control and survivor fish. Supporting our prediction, we found that the highest parasite-specific IgT responses were detected in the survivor gill explants, whereas the dominant IgM responses were localized in the head kidney and spleen explants (Fig. 7). Conversely, low or negligible parasite-specific IgT responses were detected in the survivor spleen and head kidney explants while very low IgM responses were measured in gill explants (Fig. 7).

### pIgR in the gill of trout

**Figure 8:**
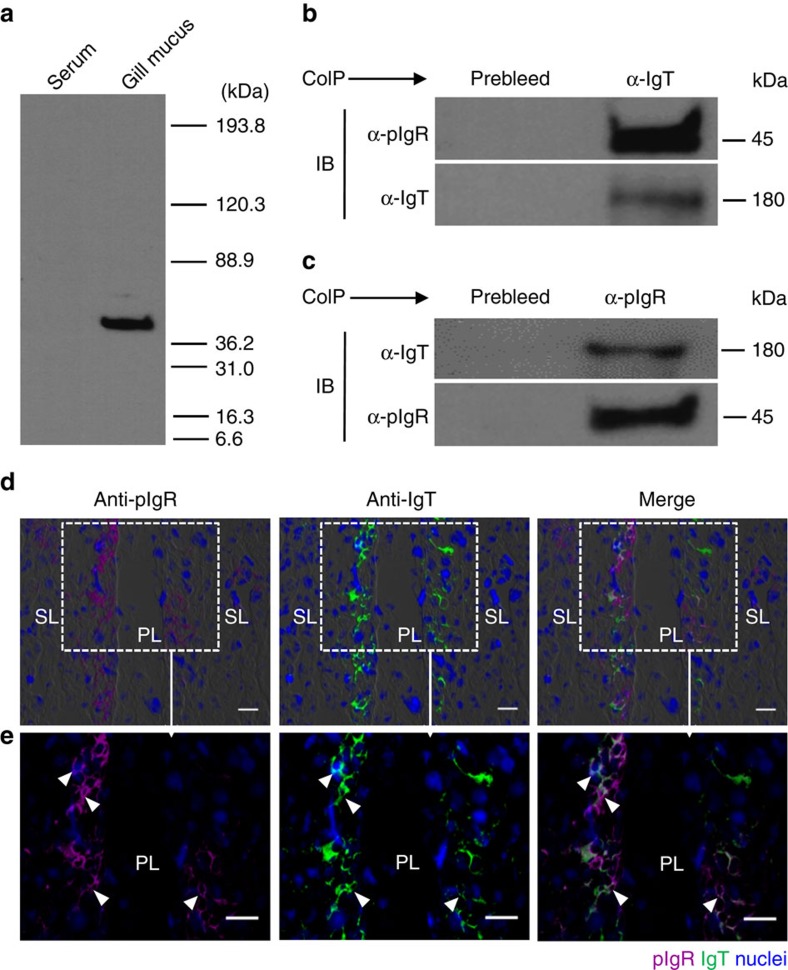
Trout pIgR associates with gill sIgs. (**a**) SDS–PAGE under reducing conditions of trout serum and gill mucus (∼5 μg each), followed by immunoblot analysis using anti-trout pIgR antibody. (**b**) Co-immunoprecipitation (CoIP) of gill mucus with anti-trout IgT antibody, followed by immunoblot analysis (IB) under reducing conditions (pIgR detection, upper panels) or non-reducing conditions (IgT detection, lower panels). (**c**) CoIP of gill mucus with rabbit anti-trout pIgR followed by IB under non-reducing conditions (IgT detection, upper panels) and reducing conditions (pIgR detection, lower panels). IgG purified from rabbit's serum before immunization (Pre-bleed) served as negative control for rabbit anti-trout pIgR and rabbit anti- trout IgT, respectively (left lane on each panel for **b**,**c**). (**d**) Immunofluorescence staining for pIgR with IgT in gill cryosections of rainbow trout. Differential interference contrast images of gill cryosections were stained with anti-trout pIgR (magenta), anti-trout IgT (green) and DAPI for nuclei (blue) (*n*=9). (isotype-matched control antibodies for anti-pIgR in Supplementary Fig. 4c online) (**e**) Enlarged sections of the areas outlined in **d** without DIC showing some pIgR/IgT colocalization (white arrowhead). Scale bars, 20 μm. Primary lamellae (PL) and secondary lamellae (SL) are shown. Data are representative of at least three independent experiments.

One important feature of sIgA and sIgM in mammals is their transport to mucosal surfaces by the polymeric immunoglobulin receptor (pIgR)^32^. We have previously found that the secretory component of trout pIgR (tSC) is associated to IgT and IgM in the gut and skin mucus, respectively^8^,^9^. In this study, the tSC was detected in gill mucus but not in the serum (Fig. 8a). To assess whether tSC was associated to gill mucus IgT, IgM and IgD, we carried out co-immunoprecipitation assays in gill mucus using antibodies against tSC (tpIgR) and immunoglobulins. Our results showed that antibodies against trout IgT, IgM and IgD were able to co-immunoprecipitate tSC in gill mucus (Fig. 8b for IgT, Supplementary Fig. 10a,b for IgM and IgD). Moreover, IgT, IgM and IgD in the gill mucus also could be immunoprecipitated by the anti-tpIgR antibody (Fig. 8c for IgT, Supplementary Fig. 10a,b for IgM and IgD). Using immunofluorescence microscopy, most of the pIgR-containing cells were observed in the primary gill lamella, while a few pIgR-positive cells were located in the secondary gill lamella. We also noticed that some of those pIgR-containing cells were associated with IgT staining (Fig. 8d,e, isotype-matched control antibody in Supplementary Fig. 4c).

## Discussion

Specialized gas-exchange structures are constantly exposed to the environment and the pathogens therein. Both invaginated (that is, lungs) and exvaginated (that is, gills) respiratory organs are separated from their environment by a mucosal epithelium, which is likely to have been subjected to comparable selective forces throughout evolutionary time. Thus, we hypothesized that respiratory mucosal tissues in both primeval and modern bony vertebrates evolved similar molecular mechanisms for mucosal adaptive immunity. This study was therefore undertaken to examine which B-cell and immunoglobulin responses play a key role in controlling pathogens and the microbiota in the gill of rainbow trout, a teleost fish model representing one of the oldest living bony vertebrate species.

In this study, we provide the first structural and functional characterization of all teleost immunoglobulins, including secreted IgD (sIgD), IgM (sIgM) and IgT (sIgT) at a mucosal surface of a teleost fish. We found that sIgT is mainly polymeric in the gill mucus, as previously shown for gut and skin sIgT^8^,^9^. In contrast, sIgD was in monomeric form as previously reported for serum IgD^12^. Overall, the amount of sIgT in gill mucus was found to be approximately fivefold higher and approximately fourfold lower than that reported in the skin and gut mucus, respectively^8^,^9^. Thus, as in mammals^33^,^34^,^35^, mucosal immunoglobulin concentrations vary at individual mucosal surfaces in fish. In addition, we found that the ratios of IgD/IgM and IgT/IgM in gill mucus were 10-fold and 100-fold higher, respectively, than those found in trout serum. The significantly higher IgT/IgM ratio in the gill mucus suggested a potential role for IgT in gill immunity.

The presence of all three trout immunoglobulins in the gill mucus implied a transport mechanism from the epithelium into the gill luminal area. Similar to sIgA and sIgM in the mammalian respiratory tract^36^, we observed that the putative tSC was associated with sIgT, sIgM and sIgD in gill mucus. Furthermore, we found that tpIgR was mainly expressed in the epithelial cells at the wall of the gill primary lamella, thus implying a role for gill tpIgR in the transport of all three immunoglobulins into the gill mucus. Notably, this is the first description of a pIgR implicated in IgD secretion in a vertebrate respiratory tract, since in mammals the receptor responsible for IgD secretion in the lung lumen is still unknown^37^. While the lack of a J chain in mammalian IgD suggested that it would not be transported by the pIgR, all trout immunoglobulin classes as well as amphibian IgX (a dedicated mucosal immunoglobulin that is secreted into the gut lumen) also do not associate with J chain^38^. In light of this data, it is tempting to hypothesize that mammalian IgD may use pIgR for its transport into the lung lumen. Although the mechanisms of interaction of trout mucosal IgM, IgD or IgT with tpIgR in the absence of J chain are currently unclear, a recent theoretical model predicts that interaction of IgT to tpIgR can occur in the absence of a J chain and would be mediated by the binding of domains 1 and 5 of tpIgR with the constant domain 3 of the IgT heavy chain^39^.

In addition to being required for the transport of sIgA and sIgM into the lung lumen, the SC is also implicated in anchoring mucosal antibodies in mucus, ensuring immune exclusion at mucosal surfaces^40^,^41^,^42^. Indeed, sIgA plays a key role in immune exclusion, as sIgA-coated bacteria are prevented from translocating from the luminal mucus into the epithelium, thereby avoiding potential systemic infections. To determine if mucosal immunoglobulins mediated immune exclusion in the teleost gills, we analysed the capacity of each gill immunoglobulin to associate with microbiota. Notably, a large population of bacteria in the gill mucus was predominantly coated by sIgT and, to a much lesser degree also by sIgM and sIgD. These data parallel that reported for trout gut, skin and nose microbiota^8^,^9^,^10^, thus further highlighting that sIgT is the main immunoglobulin interacting with microbiota at all teleost mucosal surfaces. While at a significantly lower level than that of sIgT, we also demonstrate that teleost sIgD can coat microbiota. Whether this capacity is conserved in sIgD from mammals or other vertebrates, remains to be investigated. Together, our findings represent the first identification of microbiota recognition by a specialized mucosal immunoglobulin in the gas-exchange surface of a vertebrate species. Although the lungs of mammals were thought to be sterile and devoid of microbiota^43^, the presence of a diverse microbiota has recently been confirmed in the lungs of human^44^ and mice^45^. Based on our findings suggesting a key role for sIgT in microbiota homeostasis in the gills of teleosts, it seems reasonable to predict that sIgA may also play a key role in the homeostasis of the lung microbiota. In fact, we propose that since the gill is an evaginated respiratory surface, it offers an excellent model to study the role of mucosal immunoglobulins with regard to respiratory microbiota homeostasis, since it allows for easy and clean evaluations of both the microbiota and IgT. In contrast, in mammals, studies of the lung microbiome are often confounded by potential contamination/carry-over of the upper airway microbiome into the lower airways^44^,^46^.

To date, gill immunoglobulin and B-cell responses have been poorly studied. Thus far, the only reported pathogen- or antigen-specific antibody responses in the gill pertain to the IgM class. In the catfish, Ich-specific IgM responses have clearly been shown^30^,^47^. Interestingly catfish is a teleost species that appears to lack IgT. In rainbow trout, IgT- and IgM-coated Ich trophonts have been observed in the gills of immune fish, and IgT and IgM transcript levels were moderately increased in the gills of immune fish after challenge with Ich^48^,^49^. This suggested that IgT and IgM responses against the parasite may occur in the gills. Based on these data, we chose this parasite to evaluate the specific contribution of fish immunoglobulin isotypes in mediating mucosal responses against the parasite in the gills. Importantly, our study has addressed for the first time the detection of pathogen-specific titres of all three existing teleost immunoglobulins in a mucosal tissue. Overall, we found that strong specific IgT immune responses against the parasite were specifically detected in the gill mucus, while parasite-specific IgM responses were almost exclusively detected in the serum. Moreover, studies performed with a bacterial pathogen (*F. colmunare*) supported the results obtained with the parasite, thus establishing further the prevalent induction of pathogen-specific IgT and IgM responses in the gill mucus and serum, respectively. Thus, similar to what we have previously reported for the skin and gut mucosa^8^,^9^, these findings demonstrate that IgT responses are confined to mucosal surfaces while IgM responses are systemic (that is, serum responses). Importantly, it is worth noting that this represents the first study in which a bacterial pathogen is shown to induce dominant IgT responses in a fish mucosal site, thus suggesting that IgT is probably induced by a variety of pathogens in addition to parasites. In contrast to recent reports hypothesizing a role for IgD in the gills^12^,^16^,^24^, our findings suggest that this antibody is not involved in pathogen-specific immunity in the gill mucosa. Moreover, we were unable to detect a recently reported B-cell subset expressing surface IgD, but devoid of surface IgM^16^ in either naive, infected or survivor fish. Thus, it would appear that the only role of IgD in the gill mucosa is the recognition and coating of a small percentage of the microbiota. However, at this point we cannot exclude the possibility that different pathogens or stimulation routes from the ones used here may induce relevant IgD responses in the gill or other mucosal or systemic compartment. Overall, the gill IgT responses to pathogens and microbiota described here recapitulate those found in the skin and gut mucosa. However, we detect both the highest pathogen-specific IgT titres and the greatest percentages of microbiota coated by IgT in the gut^8^ and gills. In the skin, the lower mucosal IgT titres and lower percentage of coated microbiota correlate with the lower percentages of IgT^+^ B cells and lower concentrations of skin IgT in naive fish when compared with what was found in gill and gut^8^,^9^. This may indicate that potency of the IgT responses in fish mucosal areas is related to the relative abundance of IgT^+^ B cells in those sites. Moreover, the steady-state levels of IgT^+^ B cells in these mucosal tissues may depend on the composition and/or abundance of their microbiota since in mammals it is well-known that the production of IgA and number of IgA^+^ B cells in the gut is dependent on the abundance of microbiota^50^. Indeed, important differences exist in the diversity and composition of the microbiota of trout mucosal surfaces, including the gut, gills, nose and skin^51^. Hence, it is conceivable that differences in the microbiota in the different mucosal surfaces may drive the steady-state abundance of IgT^+^ B cells, which in turn may modulate the potency and repertoire of IgT responses in that mucosal area. Further work is clearly warranted to confirm this hypothesis.

The overwhelming prevalence of IgT versus IgM titres detected in the gill mucus was reflected in the degree of IgT coating on the gill parasites, which was much higher than that seen for IgM. In contrast, previous studies in the skin and gut mucosa showed that pathogens were selectively coated by IgT with negligible IgM^8^,^9^. The minor but consistent staining of many parasites by IgM is probably due to the low but detectable local production of parasite-specific IgM in the gill of survivor fish. Alternatively, it is possible that microlesions produced by the parasite may result in leakage of plasma from damaged microvessels, thus enabling specific IgM from plasma to bind the parasite. Interestingly in mammals, both IgA and IgG play an important role in the lung mucosal immune responses, whereas in the upper respiratory tract and all other inductive sites, IgA is the main player^52^.

The high titres of pathogen-specific IgT detected in gill correlated with large accumulations of IgT^+^ B cells in the same animals. We have previously shown similar increases in IgT^+^ B cells in the skin and gut of fish that have survived Ich and *Ceratonova Shasta* infections, respectively^8^,^9^. Whether such IgT^+^ B-cell accumulations are the result of local B-cell proliferation or migration from other lymphoid organs remains a critical question. To determine if the Ich-specific IgT^+^ B-cell response occurred locally, we developed innovative *in vivo* proliferation assays and gill explant cultures. Here we show for the first time potent local proliferative IgT^+^ B-cell responses and pathogen-specific IgT production in the mucosal-associated lymphoid tissues (MALT) of a fish species. Such responses strongly suggest that accumulation of IgT^+^ B cells in the gills is due to their local proliferation, rather than migration from other organs. In further support of localized proliferation, IgT^+^ B-cell proliferative responses were not detected in the head kidney or the spleen of the same fish. Since mucosal B cells proliferate at the inductive sites in mammals, our results imply that the gills are inductive sites for the generation of IgT mucosal immune responses. In addition, the production of high titres of Ich-specific IgT in gill explant cultures, confirmed the local production of Ich-specific IgT in gill tissue. In which specific area of the gill the immune response develops, remains to be determined. In recent years a novel gill tissue, the interbranchial lymphoid tissue (ILT) has been described and shown to be a rich T-cell area, although low transcripts levels of IgM and IgT have also been detected within this tissue^53^. Due to the high likelihood that the pathogen-specific gill IgT responses observed in this study are T-cell dependent, it is conceivable that at least a portion of local B–T-cell interactions involved in this response may have occurred within or nearby the ILT. However, these B–T-cell interactions may also occur in other areas where IgT^+^ B cells proliferate, as shown in this study. Future studies are warranted to determine the importance of the ILT in pathogen-specific IgT responses in the gill.

**Figure 9:**
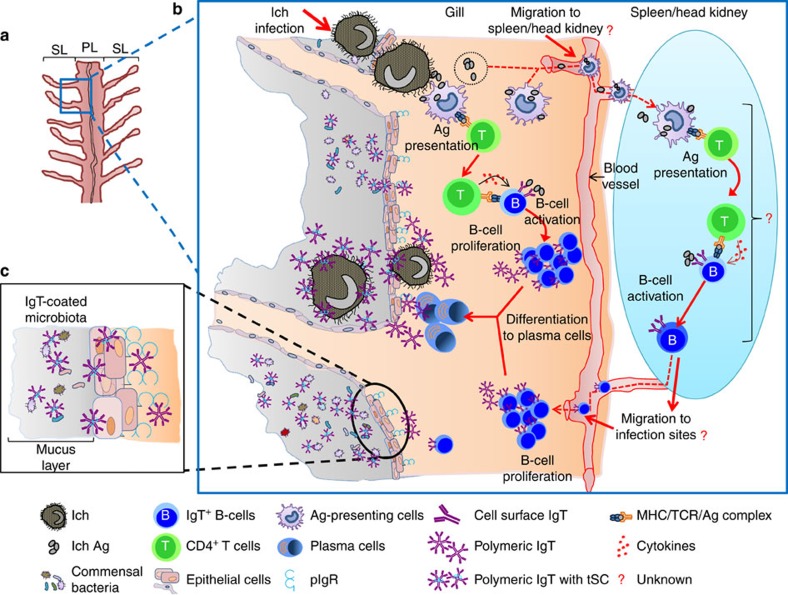
Proposed model of local IgT and IgT^+^ B-cell induction in the gills. (**a**) Scheme of a typical teleost gill filament displaying the primary (PL) and secondary (SL) lamellae. (**b**) Induction of local IgT responses in the gill based on our findings. On Ich infection, Ich antigen (Ag) are taken up by antigen-presenting cells (APC) and presented to naive CD4-T cells. B cells are then activated by Ag-specific CD4-T cells and start proliferating. Activated B cells may differentiate further into plasmablasts or plasma cells to produce large amounts of parasite-specific IgT that recognize Ich within the gill PLs or the Ich present in the mucus. We cannot rule out the possibility that on antigen uptake, loaded gill APCs may migrate into central secondary lymphoid organs (that is, spleen or head kidney) where the resulting activated IgT^+^ B cells may then home into the gill. Alternatively, since gills are highly vascularized, antigens from parasite debris may travel from the gills into the lymphoid tissues through the vascular system, where they could be taken up by systemic APCs to initiate an immune response. In the potential scenarios (as indicated by a ?) where immune responses develop in the spleen or head kidney, we propose that IgT^+^ B-cell proliferation does not occur in the spleen or head kidney because in Supplementary Fig. 9 we do not detect differences in IgT^+^ B-cell proliferative responses between control and survivor fish. This would suggest that if activated IgT^+^ B cells are generated in the spleen or head kidney, then they are probably imprinted to home into the gills where they may then proliferate. (**c**) IgT produced by IgT-secreting B cells is transported from the epithelium into the mucus layer (grey colour) by the tpIgR. The microbiota contained in the mucus is predominantly coated by sIgT.

Together, our data support a model in which on parasite infection, antigen-presentation, as well as B-cell activation and proliferation occurs within the gills, leading to the production of parasite-specific IgT (Fig. 9). At this point, we cannot exclude the possibility that on gill infection, parasite antigens are taken up by gill antigen-presenting cells (APCs) which then migrate into systemic lymphoid tissues (that is, head kidney and/or spleen). At that point, activated IgT^+^ B cells generated systemically would be imprinted to migrate into the gill where they would proliferate and turn into plasmablast or plasma cells to produce parasite-specific IgT. Alternatively, since gills are highly vascularized, antigens from parasite debris could travel from the gills into the lymphoid tissues through the vascular system, where they could be taken up by systemic APCs to initiate an immune response. Future work is warranted to address further the detailed mechanisms underlying the local production of IgT-specific responses in the gill. Finally, it is worth noting that proliferating IgT^+^ B cells in the gills appear to be randomly distributed either alone or in small groups. This would suggest the absence of organized lymphoid structures in this tissue (that is, lymph nodes), which is in agreement with the already well-known lack of organized lymphoid structures in teleost fish^7^,^54^.

In conclusion, we have identified a previously unrecognized key role of the IgT system in mucosal immune responses to pathogens and microbiota in the gill of a teleost fish. More critically, we show the generation of local IgT^+^ B-cell proliferative and pathogen-specific IgT responses in the gills, thus providing the first demonstration of locally induced B-cell and secretory immunoglobulin responses in the mucosa of a non-tetrapod species. Moreover, we show for the first time that the microbiota present within the main respiratory organ of a vertebrate is recognized by a dedicated mucosal antibody class. Thus, our findings indicate that the recognition of pathogens and microbiota by specific mucosal immunoglobulins in specialized respiratory surfaces forms part of a conserved immune strategy that predates the emergence of tetrapod species. In addition, our results reinforce the formerly stated view^26^ that specialized mucosal immunoglobulins (that is, IgA, IgT) of all vertebrate MALTs function under the guidance of primordially conserved principles. Since many fish pathogens enter the host through the gill mucosa, our findings also have special relevance from an applied perspective as they may lead to the development of fish vaccines that have the capacity to induce gill IgT mucosal immune responses.

## Methods

### Fish maintenance

Rainbow trout obtained from Limestone Springs Fish Farm were maintained in the laboratory of J.O.S. as described^8^. Fish were acclimatized for 14 days at 15 °C in an aerated aquarium with internal biofilters and fed daily with dry pellets at 1% biomass. All animal procedures were approved by the Institutional Animal Care and Use Committees of the University of Pennsylvania.

### Ich parasite isolation and infection

For Ich parasite isolation, heavily infected rainbow trout were killed by an overdose of MS-222 and placed in a container with water to allow trophonts and tomonts exit the host. After 4 h, the fish were removed, and the trophonts were left in the water at 15 °C for 1–2 days to let tomocyst formation and subsequent theront release. For parasite infection, two different sets of experiments were performed. On one hand, a group of fish was infected with a single dose of ∼5,000 theronts per fish added directly into the aquarium, and tissue samples and fluids (serum and gill mucus) were taken after 25 days (infected fish). On the other hand, a group of fish was monthly exposed over a 3-month period with ∼10,000 theronts per fish and tissue samples and fluids (serum and gill mucus) were taken 1 month after the last challenge (survival fish). Both sets of experiments were performed at least three times. For each one, the same numbers of control fish were maintained in a similar tank but without parasites.

### *F. columnare* infection

Two types of challenges with *F. columnare* were performed. In the first one (infected group), 20 fish (∼40 g per fish) were exposed by bath with a dose of 10 ml *F. columnare* suspension (OD=0.75 at 540 nm) in ∼4 l water for 1 h. Fish were sampled (serum and gill mucus) 3 weeks after exposure. For the second type of challenge (survivor group), 20 fish (∼40 g per fish) were exposed by bath with a dose of 10 ml *F. columnare* suspension (OD=0.75 at 540 nm) in ∼4 l water for 4 h. These fish were let to recover from infection (survivor fish). To this end, survivor fish were obtained by allowing the infected fish to recover from the disease during a period of 3 months at which point fish were sampled (serum and gill mucus). Both sets of infections were performed at least three times. Control fish (mock infected) were maintained in similar tanks, and were exposed to the same culture medium without the bacteria. Throughout this time, the fish were maintained in a flow through aquaria at 15 °C, and fed daily with dry pellets at 1% biomass.

### Collection of gill mucus and bacteria

For sampling, fish were anaesthetized with MS-222 and serum was collected and stored as described^9^. To obtain fish gill mucus, we modified the method described by Lumsden *et al.*^55^ To obtain the mucus, blood in the gills was first removed by perfusion with PBS–heparin through the heart until the gills were completely blanched. Gill arches were excised and rinsed with PBS three times to remove the remaining blood. Thereafter, gills were incubated for 12 h at 4 °C, with occasional shaking in protease inhibitor buffer (1 × PBS, containing 1 × protease inhibitor cocktail (Roche), 1 mM phenylmethylsulfonyl fluoride (Sigma); pH 7.2) at a ratio of 1 g of gill tissue per ml of buffer. The suspension (gill mucus) was collected into an Eppendorf tube, vigorously vortexed and centrifuged at 400*g* for 10 min at 4 °C to remove trout cells. To separate gill bacteria from mucus, the cell-free supernatant was thereafter centrifuged at 10,000*g* for 10 min. The resulting supernatant (containing gill mucus) was harvested, filtered with a 0.45 μm syringe filter (Millipore) and stored at 4 °C prior to use the same day, whereas the pellet (containing gill bacteria) was washed three times with PBS (pH 7.2) and resuspended for further analysis.

### Purification and LC-MS/MS analysis of gill mucus IgT

Gill mucus IgT was partially purified using an anti-IgT affinity column as previously described for gut and skin mucus IgT^8^,^9^. Briefly, a gill mucus sample pooled from several trout individuals was applied to the anti-trout IgT column equilibrated in PBS–EDTA (1 × PBS, containing 20 mM EDTA; pH 7.2). After several washes of the affinity column with PBS–EDTA, the bound IgT were eluted with 0.1 M glycine (pH 2.5) and immediately neutralized with 1 M Tris (pH 9.0). The eluted IgT fractions were thereafter concentrated. Approximately 2 μg of affinity purified trout IgT was resolved by SDS–PAGE on a 4–15% Tris-HCl Gradient Ready Gel (Bio-Rad) under non-reducing conditions and stained with Bio-Safe Coomassie (Bio-Rad). LC-MS/MS analysis of the IgT band was performed by the Proteomics Facility at the School of Medicine, the University of Pennsylvania. The resulting masses and MS/MS spectra were searched against the non-redundant NCBI database using the TurboSEQUEST Browser.

### Isolation of leucocytes from the gill and nasal cavities

The leucocytes from trout gill were obtained using a modified methodology from that used to isolate gill leucocytes in dab (*Limanda limanda*)^56^. Briefly, rainbow trout were anaesthetized with MS-222 and blood was collected from the caudal vein. Gills were extensively perfused and washed with cold PBS as described above to avoid blood contamination. Thereafter, gills were incubated in PBS–DTT (containing 0.37 mg ml^−1^ EDTA and 0.14 mg ml^−1^ dithiothreitol DTT) for 30 min with continuous shaking. Thereafter, the gill pieces were mechanically disaggregated on a 100-μm cell shredder and the cell fraction was collected. The remaining non-disaggregated gill tissue was then transferred into a 50 ml tube containing 10 ml collagenase buffer (Invitrogen, 0.15 mg ml^−1^ in Hanks with 5% foetal bovine serum FBS and 100 U ml^−1^ penicillin and 100 μg ml^−1^ streptomycin) for 2 h at room temperature with continuous shaking. Thereafter, the remaining gill pieces were mechanically disaggregated on a 100-μm cell shredder and the cell fraction was collected. The cell fractions from the above gill treatments were pooled, washed with Dulbecco's modified eagle medium (DMEM) and passed through a 100-μm nylon mesh. The resulting cell suspension was washed three times in fresh DMEM and layered over a 51/34% discontinuous Percoll gradient. After 35 min of centrifugation at 400*g*, leucocytes lying at the interface of the gradient were collected and washed with DMEM medium. The isolation of leucocytes from trout NALT was performed as described previously^10^. Briefly, leucocytes from the trout olfactory organ were isolated by mechanical agitation of both olfactory rosettes in DMEM medium (supplemented with 5% FBS, 100 U ml^−1^ penicillin and 100 μg ml^−1^ streptomycin) at 4 °C for 30 min. Leucocytes were subsequently collected, and the aforementioned procedure was repeated four times. Thereafter, the olfactory organ pieces were treated with PBS (containing 0.37 mg ml^−1^ EDTA and 0.14 mg ml^−1^ dithiothreitol DTT) for 30 min followed by enzymatic digestion with collagenase (Invitrogen, 0.15 mg ml^−1^) for 2 h at 20 °C. All cell fractions obtained from the olfactory organ after mechanical and enzymatic treatments were pooled and washed with modified DMEM. Cells were thereafter layered over a 51/34% discontinuous Percoll gradient and after 35 min of centrifugation at 400*g*, leucocytes lying at the interface of the gradient were collected and washed with DMEM medium.

### SDS–PAGE and western blot

Serum and gill mucus samples were resolved on 4–15% SDS–PAGE Ready Gel (Bio-Rad) under non-reducing and/or reducing conditions. The gels were either stained with Bio-Safe Coomassie or transferred onto Sequi-Blot PVDF membranes (Bio-Rad). For western blot analysis, the membranes were blocked with 8% skim milk and incubated with anti-trout IgT (rabbit pAb), anti-trout IgM (mouse monoclonal antibody (mAb)) or biotinylated anti-trout IgD (mouse mAb) antibodies followed by incubation with peroxidase-conjugated anti-rabbit, anti-mouse IgG (GE Healthcare) or streptavidin (Thermo Scientific Pierce). Immunoreactive bands were visualized using the HyGLO Chemiluminescent HRP Antibody Detection Reagent (Denville Scientific Products). For quantitative analyses of IgM, IgD and IgT in serum and gill mucus, western blot films were scanned and the signal strength of each band was determined by using ImageQuant TL software (GE Healthcare). Thereafter, the concentration of IgM, IgD and IgT were determined by plotting the obtained signal strength values on a standard curve generated for each blot using known amounts of purified trout IgM, IgD or IgT. Original images of the western blot analyses are shown in Supplementary Fig. 11.

### Flow cytometry

For flow cytometry studies of B cells in the gill, spleen and NALT, mouse anti-trout IgT and mouse anti-trout IgM mAbs were labelled with allophycocyanin (APC) and phycoerythrin (PE) using the Mix-n-Stain Antibody labelling kit (Biotium). Trout leucocytes (5 × 10^5^) were first stained with biotinylated mouse anti-trout IgD (1 μg ml^−1^) on ice for 40 min. After washing three times, Brilliant Violet 421 Streptavidin (1 μg ml^−1^, Biolegend), APC-labelled mouse anti-trout IgM and PE-labelled mouse anti-trout IgT (1 μg ml^−1^, each) were added to detect IgD^+^, IgT^+^ and IgM^+^ B cells, respectively. Samples were thereafter incubated on ice for 40 min and washed three times prior to flow cytometric analysis. Gill bacteria were stained with biotinylated mouse anti-trout IgD (1 μg ml^−1^) or with biotinylated mouse IgG1 (as isotype control) at 4 °C for 1 h with continuous agitation. After washing three times, Brilliant Violet 421 Streptavidin (1 μg ml^−1^), APC-labelled mouse anti-trout IgM and PE-labelled mouse anti-trout IgT (1 μg ml^−1^, each), or APC-labelled mouse IgG1 and PE-labelled mouse IgG2b (Biolegend, 1 μg ml^−1^, each) as isotype controls were added and incubated for 45 min at 4 °C. To discriminate bacteria from debris, gill bacteria were labelled with BacLight Green bacterial stain (Invitrogen), following the manufacturer's instructions. After three washes, analysis of stained leucocytes or bacteria was performed with a FACSCanto II (BD Biosciences) and FlowJo software (Tree Star).

### Gel filtration

Gel filtration to analyse the monomeric or polymeric state of Igs in trout gill mucus was performed as described previously for gut mucus^8^. Briefly, fractions containing the IgM, IgT or IgD were separated by gel filtration using a Pharmacia Superdex 200 column (GE Healthcare). The column was previously equilibrated with cold PBS, and protein fractions were eluted at 0.5 ml min^−1^ with PBS using a fast protein LC instrument with ÄKTApurifier systems (GE Healthcare). Protein elution was monitored by absorbance at 280 nm. Identification of IgM, IgD and IgT in the eluted fractions was performed by western blot analysis using anti-IgM, anti-IgD and anti-IgT antibodies, respectively, as described above. A standard curve was generated by plotting the elution volume of the standard proteins in a Gel Filtration Standard (Bio-Rad) against their known molecular weight, which was then used to determine the molecular weight of the eluted IgT, IgM and IgD by their elution volume. Original images of the western blot analyses are shown in Supplementary Fig. 11.

### Immunofluorescence microscopy studies

Cryoblocks of gill were taken using Optimal Cutting Temperature medium (Tissue-Tek). Cryoblocks were thereafter used to generate cryosections for immunohistochemistry assays. Gill cryosections, 6-μm thick, were used to perform immunofluorescence studies. For the detection of IgT^+^, IgM^+^ and IgD^+^ B cells in the gill, cryosections of tissue samples were fixed for 3 min in 10% (vol/vol) neutral buffered formalin. Background autofluorescence was eliminated by treatment of cryosections for 10 min with 0.1 M glycine, pH 2.3. For the detection of Ich parasite at the same time of IgT^+^ and IgM^+^ B cells, antibodies were labelled using the Mix-n-Stain Antibody labelling kit. We used CF555-labelled rabbit anti-trout IgT (0.6 μg ml^−1^), CF640R-labelled mouse anti-trout IgM (2 μg ml^−1^) and CF488A-labelled rabbit anti-Ich (1 μg ml^−1^) to incubate the cryosections overnight at 4 °C. For the detection of Ich parasite at the same time of IgT^+^ and IgD^+^ B cells, we used CF555-labelled rabbit anti-trout IgT (0.6 μg ml^−1^), CF640R-labelled mouse anti-trout IgD (2 μg ml^−1^) and CF488A-labelled rabbit anti-Ich (1 μg ml^−1^) to incubate the cryosections overnight at 4 °C. For detection of gill tpIgR, we used the same methodology described to stain skin tpIgR in cryosections using our rabbit anti-tpIgR^9^. As controls, the rabbit IgG pre-bleed and the mouse-IgG1 isotype antibodies were labelled with the same antibody labelling kits and used at the same concentrations. All slides were stained with DAPI (4′,6-diamidino-2-phenylindole; 1 μg ml^−1^; Invitrogen) before mounting with fluorescent microscopy mounting solution. Gill bacteria were stained as previously described in the above section and stained bacteria were cytospinned on glass slides and mounted with fluorescent microscopy mounting solution. Images were acquired and analysed using a Nikon Eclipse E600 fluorescence microscope and the iVision-Mac (v4.0.14) scientific imaging processing software (Bio Vision Technologies).

### Intracellular staining

Gill leucocytes were isolated as described above. Cells were fixed with fixation buffer (Biolegend) and washed twice with permeabilization wash buffer (Biolegend) following the manufacturer's instructions. Permeabilized cells were stained with monoclonal mouse anti-trout IgT (1 μg ml^−1^), or mouse IgG2b as isotype control (Biolegend; 1 μg ml^−1^) in permeabilization wash buffer at 4 °C for 30 min. Secondary antibody APC-goat anti-mouse IgG2b (Jackson ImmunoResearch Laboratories) was added after washing and incubated for 30 min at 4 °C. Analyses of stained leucocytes were performed by flow cytometry using a FACSCanto II and FlowJo software.

### Proliferation of B cells in the gill of trout

Survivor and control fish (∼50 g) were anaesthetized with MS-222 and i.v. injected with 200 μg EdU (Invitrogen) in 100 μl of PBS. After 24 h, leucocytes from gill, spleen or head kidney were obtained as described above, and 5 × 10^6^ cells were incubated with mAb mouse anti-trout IgM and anti-trout IgT (1 μg ml^−1^ each) on ice for 40 min. After washing three times, APC-goat anti-mouse IgG (Jackson ImmunoResearch Laboratories Inc.) was used as secondary antibody to detect IgM^+^ or IgT^+^ B cells. After 40 min incubation on ice, cells were washed three times and fixed. EdU^+^ cell detection was performed according to the manufacturer's instructions (Click-iT EdU Alexa Fluor 488 Flow Cytometry Assay Kit, Invitrogen). Cells were thereafter analysed in a FACSCanto II with Diva Software. For immunofluorescence analysis, gill cryosections were incubated overnight at 4 °C with rabbit anti-trout IgT (0.2 μg ml^−1^) and mouse anti-trout IgM (IgG1 isotype; 1 μg ml^−1^). After washing with PBS, cryosections were incubated for 2 h at room temperature with Cy3-conjugated AffiniPure Donkey anti-rabbit IgG or Cy5-conjugated-AffiniPure Goat anti-mouse IgG1 (Jackson ImmunoResearch Laboratories Inc.) at 2.5 μg ml^−1^ each. As primary control Abs, the rabbit pre-bleed and the mouse-IgG1 isotype controls were labelled with the same antibody labelling kits and used at the same concentrations. Stained cells were fixed and EdU^+^ cell detection was performed according to the manufacturer's instructions (Click-iT EdU Alexa Fluor 488 Imaging Kit, Invitrogen). Cell nuclei were stained with DAPI (1 μg ml^−1^) before mounting with fluorescent microscopy mounting solution. Images were acquired and analysed using a Nikon Eclipse E600 fluorescence microscope and the iVision-Mac scientific imaging processing software.

### Tissue explants culture

Control and survivor fish were killed with an overdose of MS-222, and blood was removed through the caudal vein to minimize the blood content in the collected organs. Thereafter, head kidney, spleen and gills were collected. Approximately 50 mg of each tissue was submerged in 70% ethanol for 1 min to eliminate possible bacteria on their surface and then washed twice with PBS. Thereafter, tissues were placed in a 24-well plate and cultured with 400 μl DMEM medium (Gibco), supplemented with 10% FBS, 100 U ml^−1^ penicillin and 100 μg ml^−1^ streptomycin, with 5% CO2 at 17 °C. After 7 days culture, supernatants were harvested, centrifuged and stored at −80 °C until further analysis.

### Binding of trout immunoglobulins to Ich

To assess whether infected and survivor fish had generated pathogen-specific immunoglobulins, we measured the capacity of IgT, IgM and IgD from serum, gill mucus or tissue (gill, spleen and head kidney) explant supernatants to bind to Ich using a pull-down assay previously described by us^9^. In short, parasites (∼100 tomonts) were preincubated with a solution of 0.5% BSA in PBS (pH 7.2) for 2 h at 4 °C. Thereafter, parasites were incubated with diluted gill mucus or serum or tissue (gill, head kidney and spleen) explant supernatants from infected, survivor or control fish for 2 h at 4 °C in a 300 μl volume. Dilutions were made with PBS containing 0.5% BSA (pH 7.2). After incubation, the tomonts were washed with PBS, and bound proteins were eluted with Laemmli Sample Buffer (Bio-Rad) and boiled for 5 min at 95 °C. The eluted material was resolved on 4–15% SDS–PAGE Ready Gel under non-reducing conditions, and the presence of IgT, IgM or IgD was detected by western blotting using the anti-trout IgT, IgM or IgD antibodies as described above. Original images of the western blot analyses are shown in Supplementary Fig. 11.

### Co-immunoprecipitation studies

We followed the same methodology previously reported by us to detect the association of tpIgR to IgT and IgM in gut mucus^9^. To detect whether polymeric trout IgT, IgM and IgD present in the gill mucus were associated to a secretory component-like molecule derived from tSC, we performed co-immunoprecipitating analysis using anti-trout IgT (pAb), anti-trout IgM (mAb) or anti-trout IgD (mAb) antibodies with the goal to potentially co-immunoprecipitate the tSC. To this end, 10 μg of anti-IgT, anti-IgM or anti-IgD were incubated with 200 μl of trout gill mucus. As control for these studies, the same amount of rabbit IgG (purified from the pre-bleed serum of the rabbit) were used as negative controls for anti-IgT. Similarly, a mouse-IgG1 isotype control (BioLegend) was used as negative controls for anti-IgM and anti-IgD. After overnight incubation at 4 °C, 20 μl of protein G Agarose (Invitrogen) was added into each reaction mixture and incubated for 1 h at 4 °C. Thereafter, the beads were washed five times with PBS, and subsequently bound proteins were eluted in Laemmli Sample Buffer (Bio-Rad). The eluted material was resolved by SDS–PAGE on 4–15% Tris-HCl Gradient ReadyGels (Bio-Rad) under reducing (for tSC detection) or non-reducing (for IgT, IgM or IgD detection) conditions. Western blot was performed with anti-pIgR, anti-IgT, anti-IgM or anti-IgD antibodies as described above. Original images of the western blot analyses are shown in Supplementary Fig. 11.

### Statistical analysis

An unpaired Student's *t*-test (Excel version 11.0; Microsoft) and one-way analysis of variance with Bonferroni correction (Prism version 6.0; GraphPad) were used for analysis of differences between groups. *P* values of ≤0.05 were considered statistically significant.

## Additional information

**How to cite this article:** Xu, Z. *et al.* Mucosal immunoglobulins at respiratory surfaces mark an ancient association that predates the emergence of tetrapods. *Nat. Commun.* 7:10728 doi: 10.1038/ncomms10728 (2016).

## Supplementary Material

Supplementary Information Supplementary Figures 1-11 and Supplementary References
