# Supplementary material for: Mucosal immunoglobulins at respiratory surfaces mark an ancient association that predates the emergence of tetrapods
Source: Nat Commun. 2016 Feb 12;7:10728. doi: 10.1038/ncomms10728 (PMC4754351; doi:10.1038/ncomms10728)
Supplement: Supplementary Information — Supplementary Figures 1-11 and Supplementary References [file ncomms10728-s1.pdf]

a

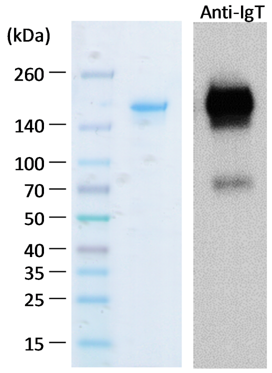

c

Peptide	Position
KNDMEVEAQTPTLGK	187-201
NDMEVEAQTPTLGK	188-201
QTDHIDLKPIESK	293-305
VSTLTIDQTK	312-321
VQCSAMKSGEDTPVIQDISFTK	328-349
SGEDTPVIQDISFTK	335-349
KGNYFVTSVFTTTK	400-423
GNYFVTSVFTTTK	401-423

b **Protein Report**

gi|58201864 (100%), 50,479.6 Da
immunoglobulin tau heavy chain secretory form, partial [Oncorhynchus mykiss]
8 unique peptides, 8 unique spectra, 16 total spectra, 74/463 amino acids (16% coverage)

1	V S T S T Q F L E A	K S L S S E D S A V	Y Y C A R H P T V T	V W A S A F D Y W G	K G T Q V T V S A A	T T A P S T L L T L
61	M N C G I P S N D I	Y I L G C V A K G F	S P S S H T F Q W T	D A S G K A L T D F	V Q Y P A V Q S G E	T Y T G V S Q L R V
121	A K N V W E N S K S	F R C S V D H P G G	A K T A V I N K P V	P K S R T V S L L S	A P I G T T Q Y L M	C M I E D F T S E T
181	V K V T W K K N D M	E V E A Q T P T L G	K R P S G L Y S G S	S L L K V I N S D W	N N K V K Y S C V V	E H Q G E T I S K T
241	T S K T E P L T V T	L N P P R V R E V F	L D N Q A V L E C V	I T A T D Q N T V S	G T N I T W H V N G	K K Q T D H I D L K
301	P I E S K G N L N S	R V S T L T I D Q T	K W T N V N K V Q C	S A M K S G E D T P	V I Q D I S F T K G	S E A P S V S V H I
361	L P E E D T K K D G	D V T L V C L V V S	P S L C D V Y I M W	K E D S G E Y Q E G	V T S P P Q K T K K	G N Y F V T S V F T
421	T T K D K W D T N V	L F T C A V K H A G	S D N S T S P K E M	S V S K S T G N S C	E D K	

Sequence Coverage	Protein	Accession	Prob	%Spec	#Pep	#Uniq	#Spec	%Cov	Weight
	immunoglobulin tau heavy chain secretory form, ...	gi|58201864	100%	0.40%	8	8	16	16%	50462

Legend:

Protein coverage

Supplementary Figure 1. LC-MS/MS analysis of trout IgT from gill mucus. (**a**) Affinity purified trout IgT from gill mucus was resolved on a 4-15% SDS-PAGE under non-reducing conditions and stained with Coomassie blue (left) or analyzed by immunoblot with a mAb anti-trout IgT antibody (right). The observed band corresponding with the reported molecular mass of IgT was subjected to in-gel digestion and LC-MS/MS analysis. (**b**) The resulting masses and MS/MS spectra were searched against the non-redundant NCBI database, showing that 8 peptides matched with the secretory form of trout IgT heavy chain (accession no. AAW66981), comprising 16% of the sequence. The amino acid sequence of the parent protein (IgT heavy chain) is shown numbered, and the protein sequence coverage is shown in highlighted yellow. (**c**) The sequences and positions of each peptide hit are shown.

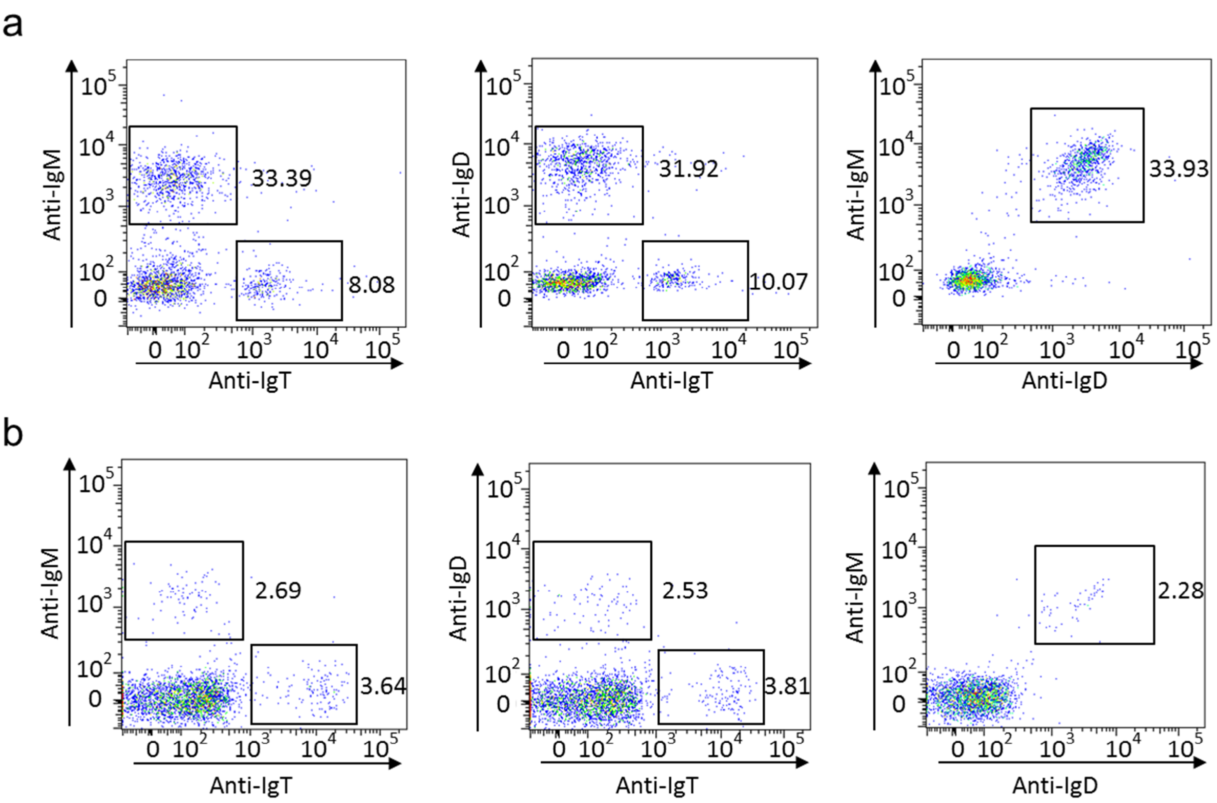

Supplementary Figure 2. Staining of B cell subsets from trout spleen and NALT.

Leukocytes from the **(a)** spleen **and (b) NALT** were stained with anti-trout IgT, IgM and IgD mAbs and analyzed by flow cytometry. Dot plots of leukocytes from spleen **and NALT**. Numbers adjacent to outlined areas indicate percentage IgT$^+$, IgM$^+$ or IgD$^+$ B cells in lymphocyte population. Data are representative of at least three independent experiments (*n* = 20). The results show the presence of a negligible frequency (~0.5±0.07%) and (~0.3± 0.09%) of IgD$^+$IgM$^-$ B cells in the lymphoid gate of spleen and NALT leukocytes respectively. Moreover, while some IgM$^+$IgT$^+$ and IgD$^+$IgT$^+$ lymphocytes could be detected in the spleen, those represented a very minor fraction 0.3±0.1% and 0.7±0.2% respectively of all lymphocytes. In the NALT these percentages are even lower, and IgT$^+$IgM$^+$ and IgT$^+$IgD$^+$ B cells represent 0.08±0.02% and 0.14±0.06% respectively of the lymphocyte gate.

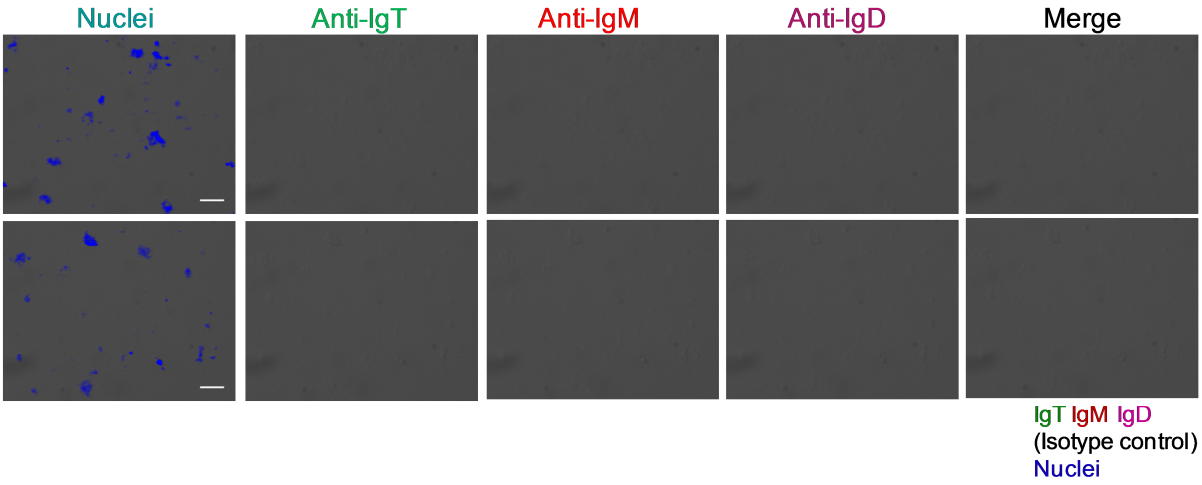

Supplementary Figure 3. Staining of trout gill bacteria with isotype control antibodies for anti-IgT, anti-IgM and anti-IgD mAbs. Differential interference contrast images (DIC) of gill bacteria stained with a DAPI-Hoeschst solution (blue), isotype control antibodies for anti-trout IgT (green), for anti-trout IgM (red), or for anti-trout IgD (magenta) mAbs, and merging isotype control antibodies for IgT, IgM and IgD staining. Scale bar, 5 µm. Upper and lower panels display two different samples, representative of at least three independent experiments.

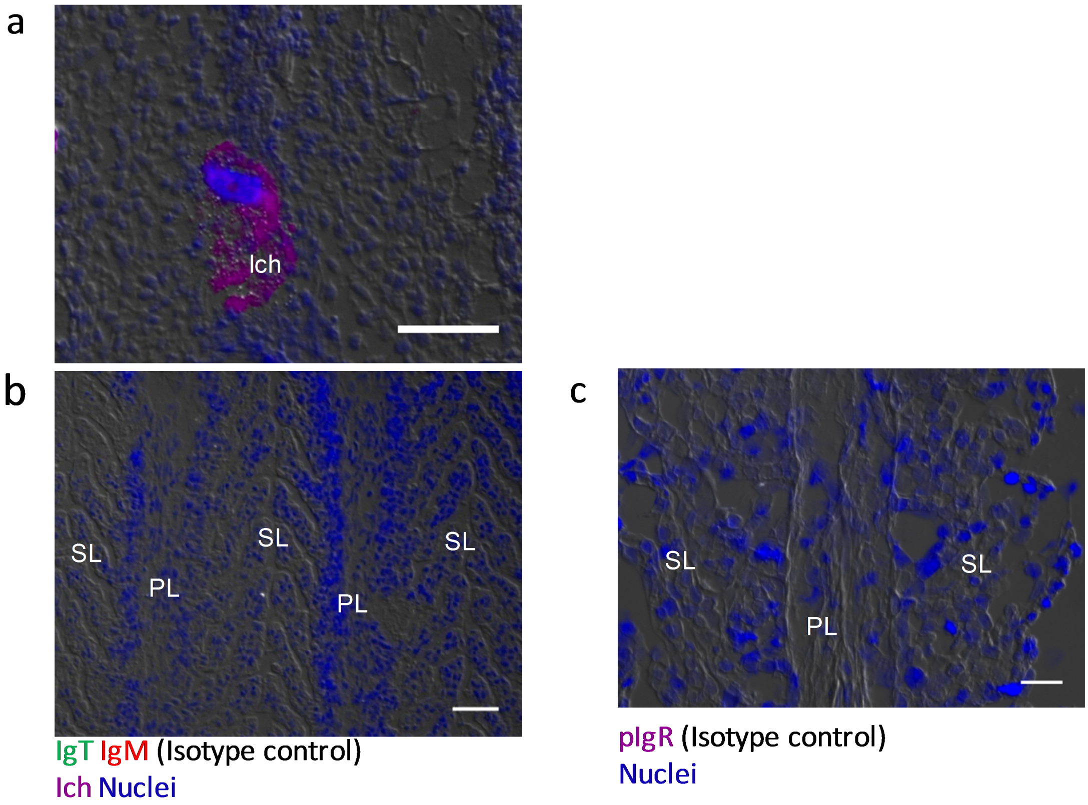

Supplementary Figure 4. Isotype control staining for anti-IgT, anti-IgM and anti-pIgR antibodies in trout gill cryosections. Differential interference contrast images of gill cryosections from 25 days Ich-infected fish (**a**), survivor fish (**b**), and control fish (**c**), with merged staining of isotype control antibodies for anti-trout IgT (green) or anti-trout IgM mAbs (red) (**a**,**b**); or for anti-trout pIgR pAb (magenta, **c**). Nuclei were stained with DAPI (blue, **a-c**) and Ich with anti-Ich pAb (magenta,**a**). Primary lamellae (PL), Secondary lamellae (SL), and Ich (Ich) are shown. Scale bars, 20 μm. Data are representative of three independent experiments.

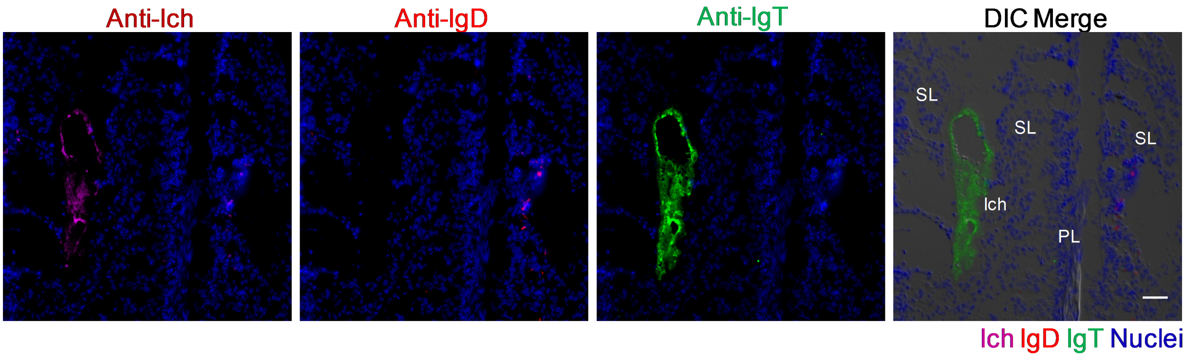

Supplementary Figure 5. Parasites are not coated by IgD in infected trout. Microscope images of slides showing immunofluorescence staining of Ich in a gill cryosection from 25 days infected fish. From left to right: Ich (magenta), IgD (red) and IgT (green) with nuclei stained with DAPI (blue); DIC images showing merged staining. Primary lamellae (PL), Secondary lamellae (SL), and Ich are shown. Scale bars, 20 μm.

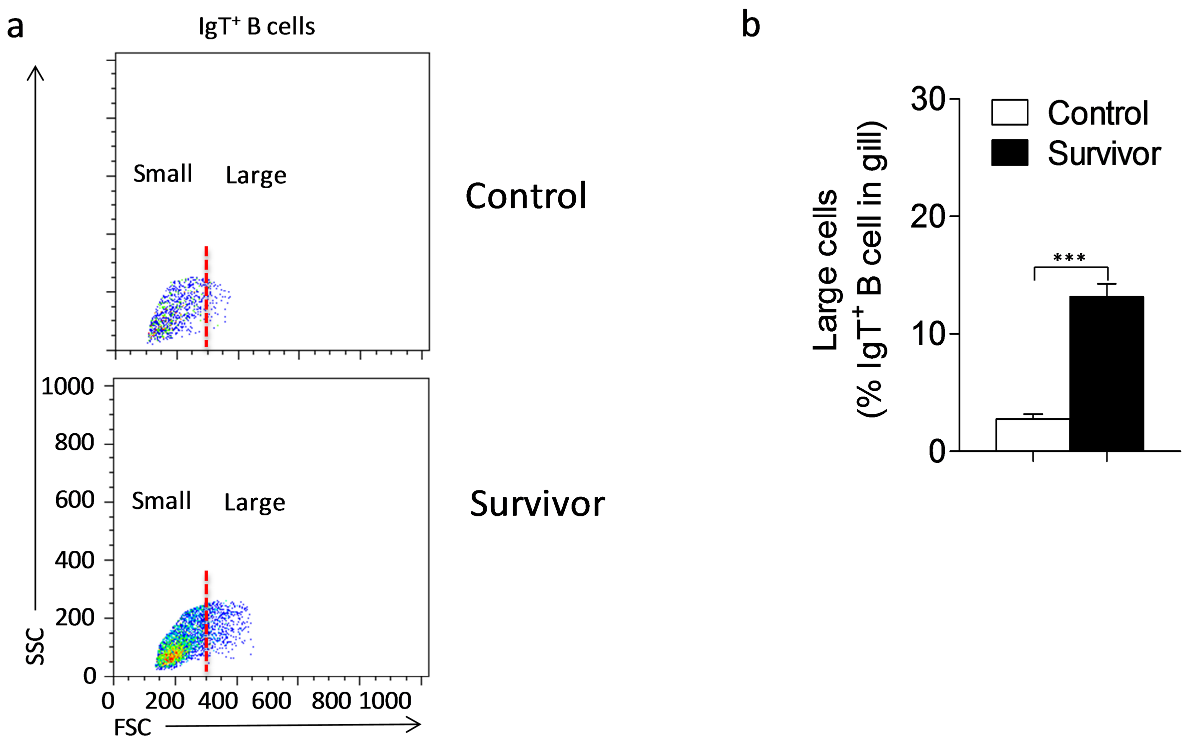

Supplementary Figure 6. Large IgT$^+$ B cell numbers increase in the gill of survivor fish. (**a**) Representative dot plot of intracellular staining of gill IgT$^+$ B cells in control fish (upper panel) and survivor fish (lower panel). FSC, forward scatter; SSC, side scatter. Data are representative of at least three independent experiments (**b**) Percentage of large IgT$^+$ B cells in total IgT$^+$ B cell population of gill from control and survivor fish. ***P<0.001 (unpaired Student's t-test). Data are expressed as mean and s.e.m. obtained from 12 individual fish per group.

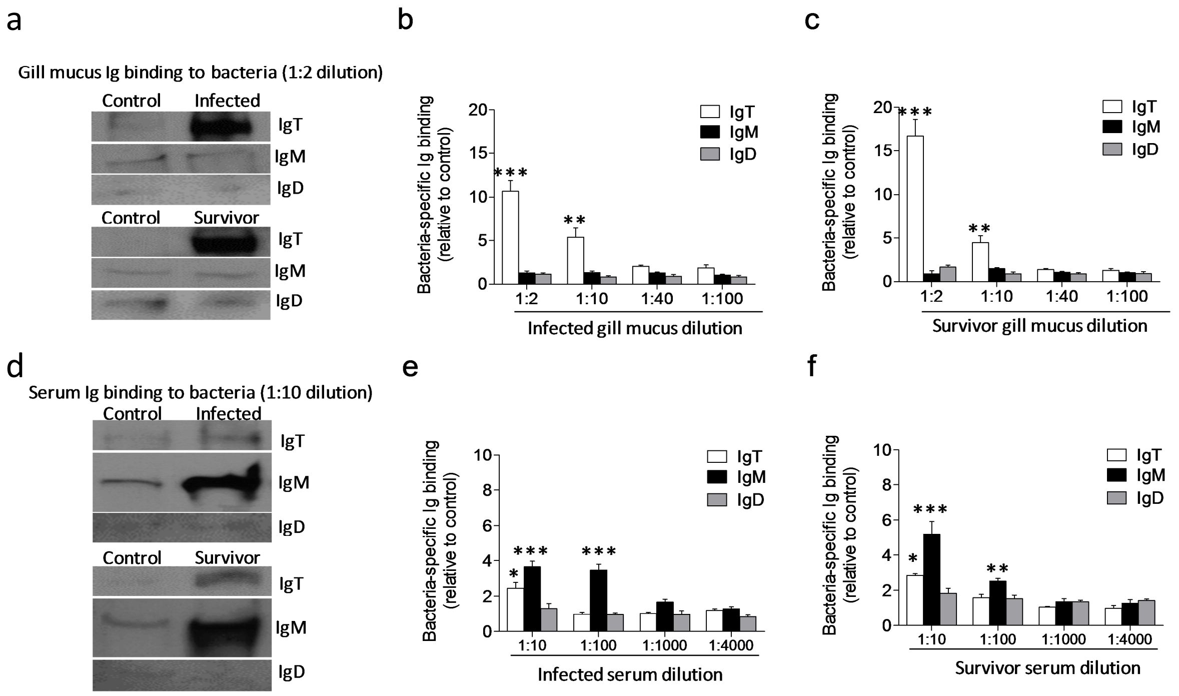

Supplementary Figure 7. Immunoglobulin responses in the gill mucus and serum of trout infected with *Flavobacterium columnare*. (**a**) Immunoblot analysis of IgT, IgM and IgD specific binding to *F. columnare* in gill mucus (dilution 1:2) from infected and survivor fish. (**b,c**) IgT, IgM and IgD specific binding to *F. columnare* in dilutions of gill mucus from infected (**b**) and survivor (**c**) fish, measured by densitometric analysis of immunoblots and presented relative to values of control fish (*n* = 12-15 per group). (**d**) Immunoblot analysis of IgT, IgM and IgD specific binding to *F. columnare* in serum (dilution 1:10) from infected and survivor fish. (**e,f**) IgT, IgM and IgD specific binding to *F. columnare* in dilutions of serum from infected (**e**) and survivor (**f**) fish, measured by densitometric analysis of immunoblots and presented relative to values in control fish (*n* = 12-15 per group). *P<0.05, **P<0.01 and ***P<0.001 (unpaired Student's *t*-test). Data are representative of at least three independent experiments (mean and s.e.m.).

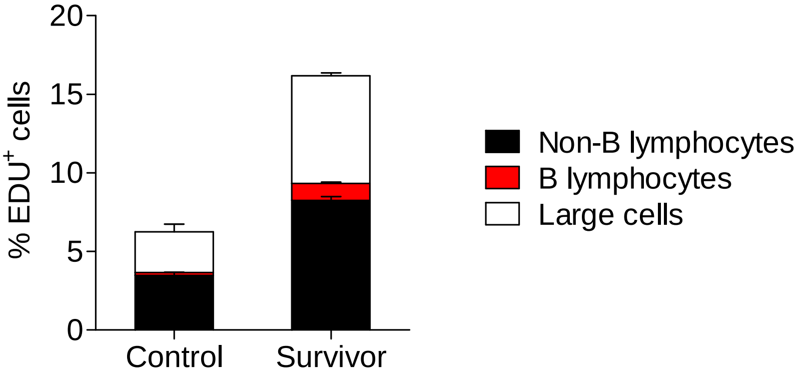

Supplementary Figure 8. Proliferation of leukocytes in gill of control and survivor fish.

Percentage of EdU^+ cells in total gill leukocytes of control and survivor fish (*n* = 12). Cell proliferation was assessed by flow cytometry as described in Fig. 6. Data are representative of at least three independent experiments (mean and s.e.m.).

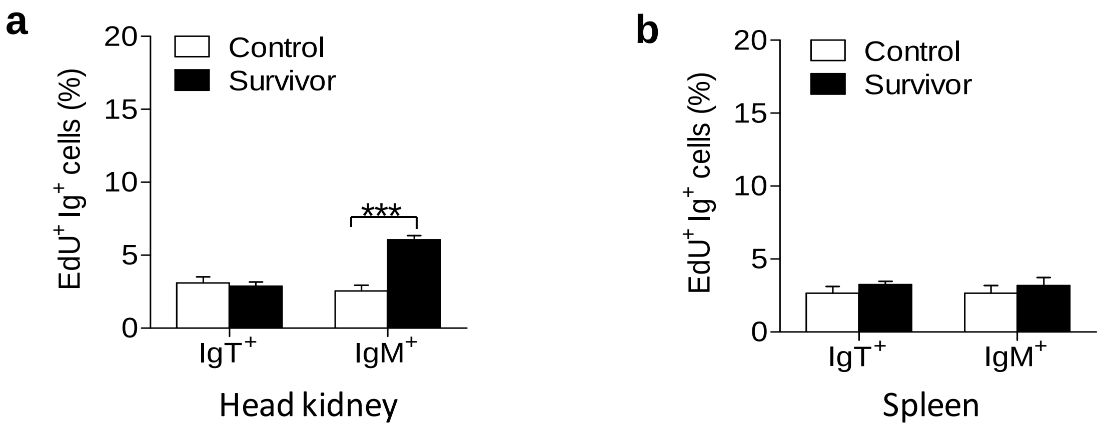

Supplementary Figure 9. Proliferation of B cells in head kidney and spleen in survivor fish. Percentage of EdU^+ cells in total IgT^+ or IgM^+ B cell populations in head kidney (**a**) and spleen (**b**) of control and survivor fish (n = 12). Data are representative of at least three independent experiments (mean and s.e.m.). Statistical analysis was performed by unpaired Student's *t*-test. ***P<0.001.

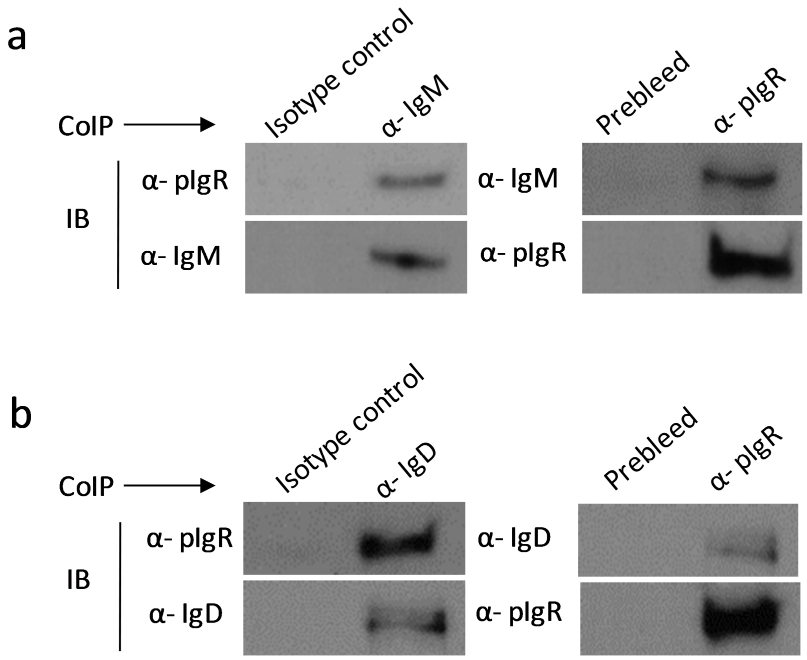

Supplementary Figure 10. Trout pIgR associates with gill sIgM and sIgD.

Coimmunoprecipitation (CoIP) of gill mucus with anti-trout IgM (**a**) or anti-trout IgD mAbs (**b**), followed by immunoblot analysis (IB) under reducing conditions (pIgR detection, upper panels, **a**,**b** left) or non-reducing conditions (**a** left, IgM detection, lower panels; **b** left, IgD detection, lower panels). CoIP of gill mucus with rabbit anti-trout pIgR pAb followed by IB under non-reducing conditions (**a** right, IgM detection, upper panels; **b** right, IgD detection, upper panels) and reducing conditions (**a**,**b** right, pIgR detection, lower panels). IgG purified from rabbit's serum before immunization (Prebleed) and mouse IgG1 served as negative control for rabbit anti-trout pIgR and mouse anti-trout IgM or mouse anti-trout IgD, respectively (left lane on each panel for **a** and **b**). This figure shows that, similar to IgT (Fig. 8), both IgM and IgD are able to associate with tpIgR. In the absence of J chain, the mechanisms of interaction of trout mucosal IgM, IgD or IgT with tpIgR are thus far unknown. In mammals, the interaction of pIgR with sIgA is partly mediated by the J chain. However, it is well-known that domains 1 and 5 of

mammalian pIgR are the main domains involved in the binding of mucosal immunoglobulins with pIgR[1]. Interestingly, teleost fish pIgR, including that of rainbow trout, contain only two domains which are homologous precisely to domains 1 and 5 of mammalian pIgR[2]. In mammals, domain 1 mediates direct binding of pIgR to IgA and J chian through non-covalent interactions, while domain 5 is involved in the covalent binding of pIgR with IgA, although a non-covalent interaction between domain 5 and IgA is thought to occur prior to the formation of the covalent disulfide bond between domain 5 and IgA is established[3,4]. It has been shown that domain 1 is very conserved across species, and that the interaction of IgA with this domain alone is sufficient for maintaining binding of pIgR to IgA[1]. Moreover, normal transcytosis of the pIgR-pIgA complex can occur under conditions in which the disulfide bonding from domain 5 with IgA is abrogated[5,6]. This strongly suggests that either binding of IgA with domain 1 is sufficient for maintaining the pIgR-pIgA complex together, or that in addition to the non-covalent forces between domain 1 and IgA, there are also non-covalent forces between domain 5 and the IgA involved in the binding between pIgR and IgA. Thus, it is conceivable that in rainbow trout, the binding between pIgR and IgT, IgM or IgD occurs in a non-covalent fashion through their interaction with domain 1 alone, or in combination with domain 5. In support of this hypothesis, a recent study has proposed a theoretical model that predicts that binding of IgT to pIgR can occur in the absence of J chain[1]. Future studies will have to be devoted to solely address this hypothesis.

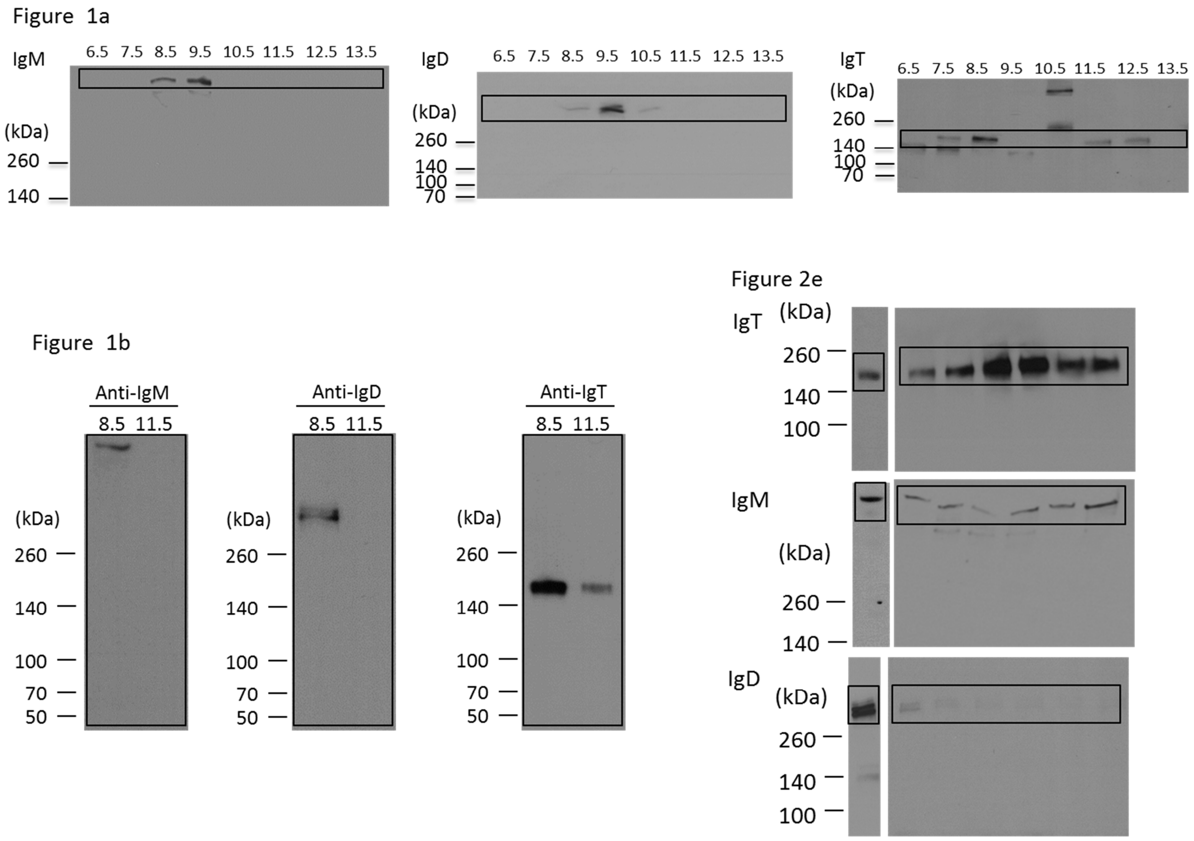

Supplementary Figure 11. Original images of the western blot analyses in the main figures.

Figure 5a

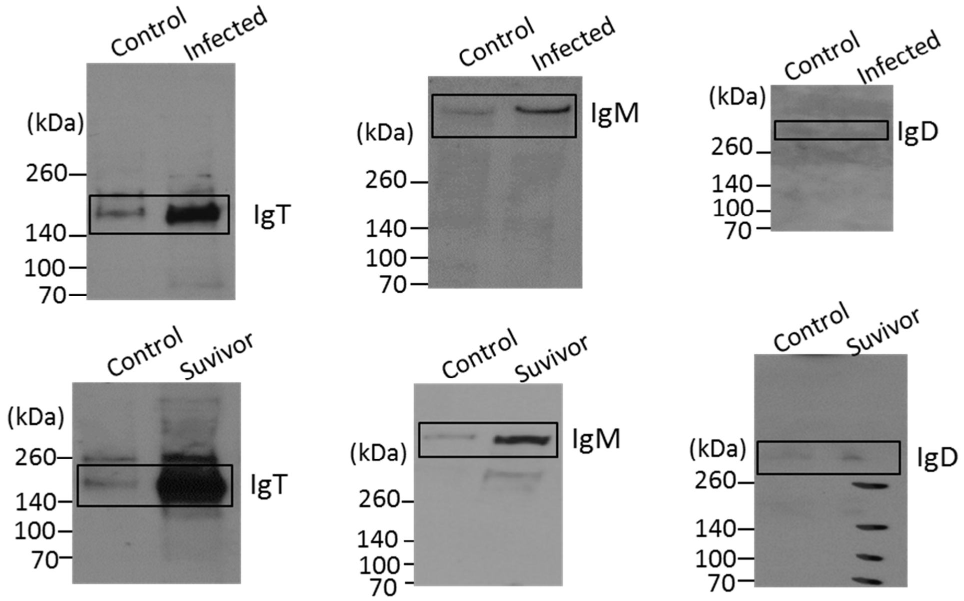

Figure 5d

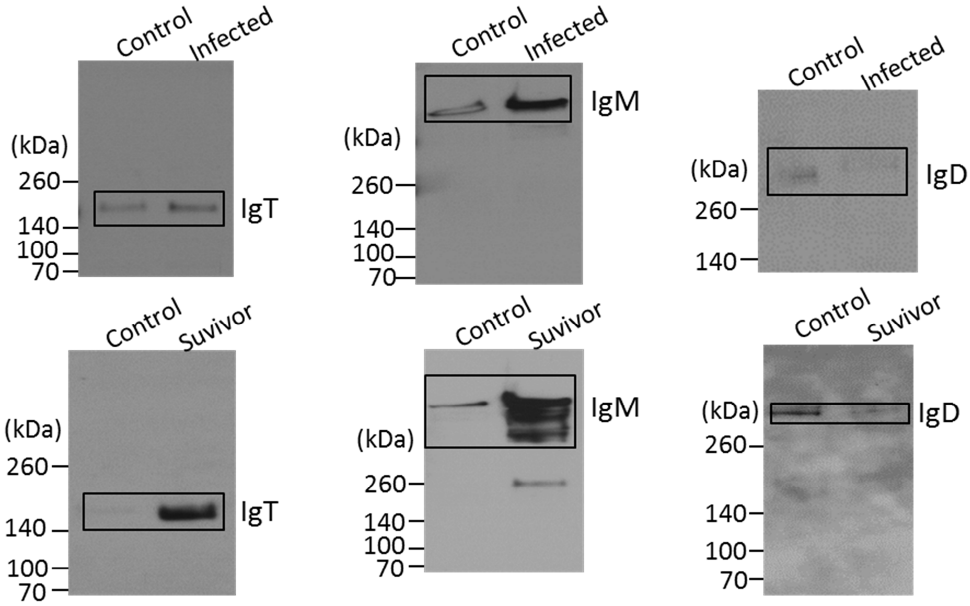

Supplementary Figure 11. Continued

Figure 7a

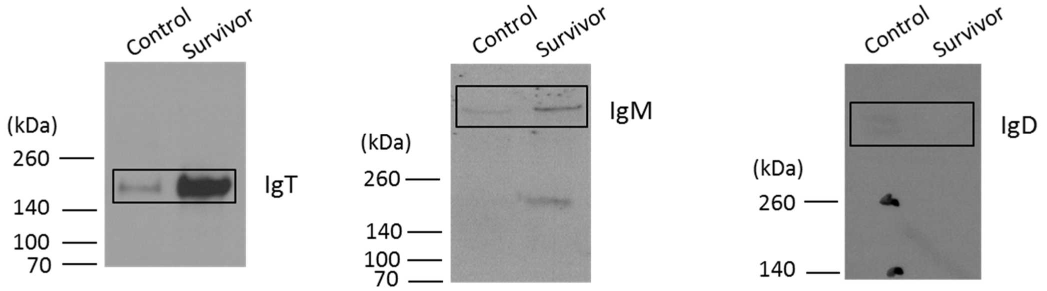

Figure 7b

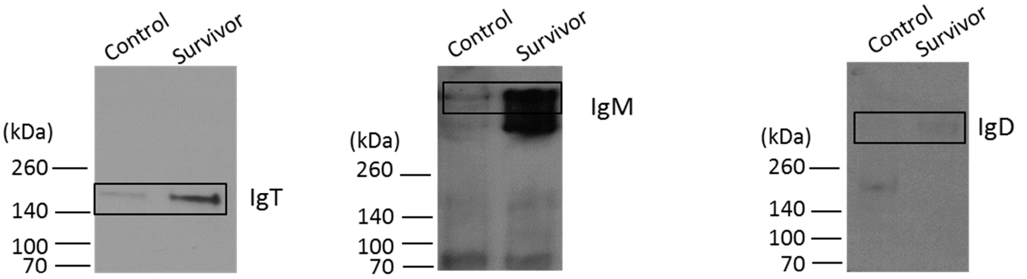

Figure 7c

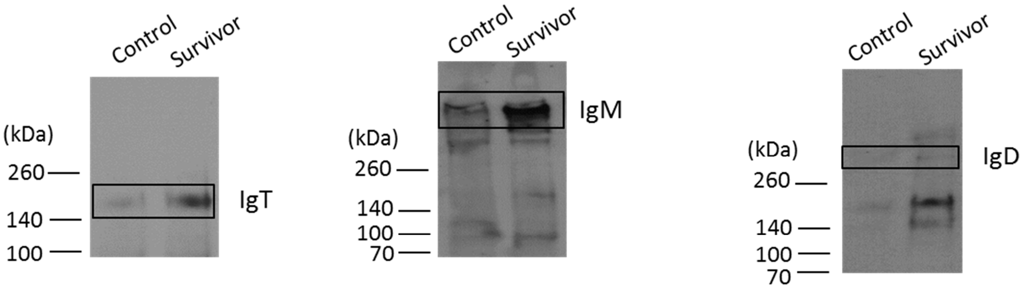

Figure 8a

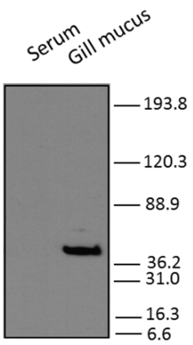

Figure 8b

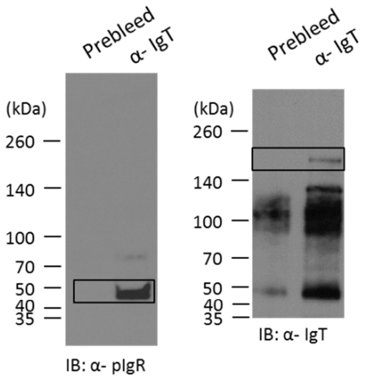

Figure 8c

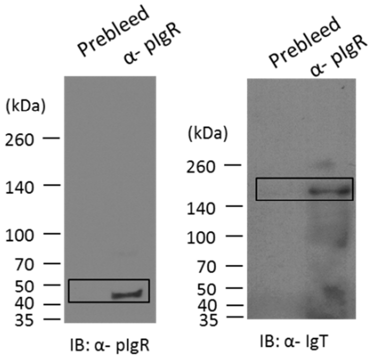

Supplementary Figure 11. Continued

Supplementary references

1 Kaetzel, C. S. Coevolution of mucosal immunoglobulins and the polymeric immunoglobulin receptor: evidence that the commensal microbiota provided the driving force. *ISRN Immunology* **2014** (2014).

2 Zhang, Y. A. *et al.* IgT, a primitive immunoglobulin class specialized in mucosal immunity. *Nat. Immunol.* **11**, 827-835 (2010).

3 Coyne, R. S., Siebrecht, M., Peitsch, M. C. & Casanova, J. E. Mutational analysis of polymeric immunoglobulin receptor/ligand interactions. Evidence for the involvement of multiple complementarity determining region (CDR)-like loops in receptor domain I. *J. Biol. Chem.* **269**, 31620-31625 (1994).

4 Brandtzaeg, P. Secretory immunity with special reference to the oral cavity. *Journal of oral microbiology* **5** (2013).

5 Chintalacharuvu, K. R., Raines, M. & Morrison, S. L. Divergence of human alpha-chain constant region gene sequences. A novel recombinant alpha 2 gene. *J. Immunol.* **152**, 5299-5304 (1994).

6 Tamer, C. M., Lamm, M. E., Robinson, J. K., Piskurich, J. F. & Kaetzel, C. S. Comparative studies of transcytosis and assembly of secretory IgA in Madin-Darby canine kidney cells expressing human polymeric Ig receptor. *J. Immunol.* **155**, 707-714 (1995).
